# Lithocholic acid alleviates fatty liver in mice and non-human primate macaques

**DOI:** 10.1093/lifemeta/loag023

**Published:** 2026-07-23

**Authors:** Qi Qu, Yan Chen, Guo Shen, Xiaojiao Huang, Fang-Han Li, Xiaojun Wang, Cixiong Zhang, Zhumei Wei, Liping Xie, Jianfeng Wu, Jinlong Yang, Qianru Xu, Nan Xu, Weiqi Xu, Su Chen, Jianping Qi, Chen-Song Zhang, Sheng-Cai Lin

**Affiliations:** Zhongzhou Laboratory for Integrative Biology, School of Basic Medical Sciences, Henan University, Kaifeng, Henan 475004, China; State Key Laboratory of Cellular Stress Biology, School of Life Sciences, Faculty of Medicine and Life Sciences, Xiamen University, Xiamen, Fujian 361102, China; State Key Laboratory of Cellular Stress Biology, School of Life Sciences, Faculty of Medicine and Life Sciences, Xiamen University, Xiamen, Fujian 361102, China; Guangxi Huaren Jiying Biotechnology Co., Ltd., Nanning, Guangxi 530000, China; Guangxi Huaren Jiying Biotechnology Co., Ltd., Nanning, Guangxi 530000, China; State Key Laboratory of Cellular Stress Biology, School of Life Sciences, Faculty of Medicine and Life Sciences, Xiamen University, Xiamen, Fujian 361102, China; State Key Laboratory of Cellular Stress Biology, School of Life Sciences, Faculty of Medicine and Life Sciences, Xiamen University, Xiamen, Fujian 361102, China; State Key Laboratory of Cellular Stress Biology, School of Life Sciences, Faculty of Medicine and Life Sciences, Xiamen University, Xiamen, Fujian 361102, China; Guangxi Huaren Jiying Biotechnology Co., Ltd., Nanning, Guangxi 530000, China; State Key Laboratory of Cellular Stress Biology, School of Life Sciences, Faculty of Medicine and Life Sciences, Xiamen University, Xiamen, Fujian 361102, China; Laboratory Animal Research Center, Xiamen University, Xiamen, Fujian 361102, China; School of Pharmaceutical Sciences, Key Laboratory of Smart Drug Delivery, State Key Laboratory of Advanced Drug Formulations, Fudan University, Shanghai 201203, China; Zhongzhou Laboratory for Integrative Biology, School of Basic Medical Sciences, Henan University, Kaifeng, Henan 475004, China; Zhongzhou Laboratory for Integrative Biology, School of Basic Medical Sciences, Henan University, Kaifeng, Henan 475004, China; Department of Hepatic Surgery, Shanghai Cancer Center, Fudan University, Shanghai 200032, China; Department of Oncology, Shanghai Medical College, Fudan University, Shanghai 200032, China; Zhongzhou Laboratory for Integrative Biology, School of Basic Medical Sciences, Henan University, Kaifeng, Henan 475004, China; School of Pharmaceutical Sciences, Key Laboratory of Smart Drug Delivery, State Key Laboratory of Advanced Drug Formulations, Fudan University, Shanghai 201203, China; Zhongzhou Laboratory for Integrative Biology, School of Basic Medical Sciences, Henan University, Kaifeng, Henan 475004, China; State Key Laboratory of Cellular Stress Biology, School of Life Sciences, Faculty of Medicine and Life Sciences, Xiamen University, Xiamen, Fujian 361102, China; Department of Gastrointestinal Surgery, Institute of Gastrointestinal Oncology, Zhongshan Hospital of Xiamen University, Xiamen, Fujian 361004, China; Zhongzhou Laboratory for Integrative Biology, School of Basic Medical Sciences, Henan University, Kaifeng, Henan 475004, China; State Key Laboratory of Cellular Stress Biology, School of Life Sciences, Faculty of Medicine and Life Sciences, Xiamen University, Xiamen, Fujian 361102, China; Department of Gastrointestinal Surgery, Institute of Gastrointestinal Oncology, Zhongshan Hospital of Xiamen University, Xiamen, Fujian 361004, China

Dear Editor,

Caloric restriction (CR) is an effective intervention for delaying aging and improving metabolic health. Recently, we identified lithocholic acid (LCA), a secondary bile acid elevated during CR, that alone can recapitulate the anti-aging benefits of CR [1, 2]. In aged mice, administration of LCA at doses that give rise to circulating concentrations comparable to those detected in mice after CR generates rejuvenating effects on muscles, including increased levels of NAD^+^, improved grip strength, and enhanced running distance and duration [1, 2]. At the organismal level, LCA extends lifespan and healthspan, as tested in *Caenorhabditis elegans* and *Drosophila melanogaster* [1]. Mechanistically, LCA binds to its receptor Tubby-like protein 3 (TULP3), which is constitutively associated with sirtuins, thereby activating these deacetylases [2]. The activated sirtuins then deacetylate the V1E1 subunit of the lysosomal v-ATPase [1, 2], leading to the inhibition of v-ATPase and subsequent activation of AMP-activated protein kinase (AMPK) via the lysosomal glucose-sensing pathway [3–5]. Importantly, the lysosomal glucose sensing-AMPK activation pathway is intrinsically triggered in response to physiologically low glucose during short-term fasting [3, 6]. The engagement of this pathway by LCA, and also by metformin [7, 8], promotes longevity by coordinating AMPK, sirtuins, and NAD^+^.

However, there are concerns regarding the benefits and safety of LCA. First, LCA has been documented to have harmful effects, predominantly hepatotoxicity, in experimental or pathological models in which LCA levels increased hundreds of fold. In mice, administering LCA via intraperitoneal injection at doses of 250 mg/kg (mpk) per day [9–11], or dietary supplementation with 1% (w/w) LCA [12–14], can induce cholestasis, bile duct obstruction, and liver damage. In isolated hepatocytes, LCA has also been reported to promote cell death [15–17], or cause DNA damage when combined with other genotoxic agents [18]. Moreover, recent work has suggested that LCA can promote the progression of hepatocellular carcinoma (HCC) [19]. These findings raise concerns about what dosage of LCA can maintain be­nefits without causing hepatotoxicity in animals of various ages, particularly given that aged animals are more susceptible to bile acid-induced liver injury [20–23]. Additionally, it is uncertain whether the beneficial effects of LCA observed in mice also hold true for primates, including humans [24, 25].

We sought to assess the dosage and safety (specifically the risk of hepatotoxicity) of LCA while evaluating its efficacy in reducing fatty liver, a condition associated with aging and obesity-related metabolic dysfunctions, and one that can also be alleviated through CR. These assessments were performed in mice, and also in non-human primate macaques (*Macaca fascicularis*). The mice were first fed with high-fat diet (HFD) for 5 months to induce fatty liver and obesity, and then administered with LCA via drinking water at 1 g/L (using 2-hydroxypropyl-β-cyclodextrin as a solvent; Qu et al. [1]), which yielded LCA concentrations in the serum of approximately 1 μmol/L (Fig. 1a), close to the levels found in calorie-restricted mice [1, 2]. Similar exposure of LCA was detected in the livers of these mice (1.69 μmol/L or 1.59 nmol/g liver; Fig. 1b and c). As seen previously in the muscle tissues of non-obese mice [1, 2], LCA robustly activated AMPK in the livers of the obese mice, as evidenced by increased phosphorylation of AMPKα at Thr172 and its substrate, acetyl-coenzyme A carboxylase (ACC), at Ser79 (Fig. 1d). After 4 weeks of LCA treatment, we found marked alleviation of fatty liver in these mice, as determined by measuring hepatic triglyceride (TAG) content (Fig. 1e and j) and by histological analyses of liver sections (hematoxylin and eosin [H&E] staining; Fig. 1f and k). LCA also reduced the mass of white adipose tissues (Supplementary Fig. S1a), albeit no reduction in the overall mouse body weight (Supplementary Fig. S1b). Furthermore, LCA improved the systemic metabolic dysfunctions associated with fatty liver and obesity, as demonstrated by enhanced glucose tolerance (measured by an oral glucose tolerance test [OGTT]; Fig. 1g), improved insulin sensitivity (insulin tolerance test [ITT]; Fig. 1h), and increased glucose infusion rate (GIR) and stronger inhibition of hepatic glucose production (HGP) during the hyperinsulinemic-euglycemic clamp (Fig. 1i). To determine whether these beneficial effects depend on hepatic AMPK, we used mice with liver-specific knockout of *AMPKα* (both *AMPKα1* and *AMPKα2*; *AMPKα*-LKO, validated in Supplementary Fig. S1c). We found that this hepatic-specific knockout of *AMPKα* virtually abolished the effect of LCA on hepatic TAG contents (Fig. 1j and k), glucose tolerance, and insulin sensitivity (Fig. 1l and m), compared to wild-type (WT) mice. Additionally, given that oral LCA stimulates glucagon-like peptide-1 (GLP-1) secretion in mice [1] and that GLP-1 can help alleviate fatty liver, we measured plasma GLP-1 levels in LCA-treated obese mice, and found that LCA led to an increase in GLP-1 levels (Supplementary Fig. S1d). In addition, treatment with 1 μmol/L LCA significantly reduced intracellular TAG content and lipid droplet size in oleic acid (OA)-pretreated primary mouse hepatocytes, as well as in primary-like human HepaRG and human hepatoma HepG2 cells (Fig. 1n and o; Supplementary Fig. S2a–h). All lipid-lowering effects were no longer observed in *AMPKα* deletion (Fig. 1n and o).

**Figure 1:**
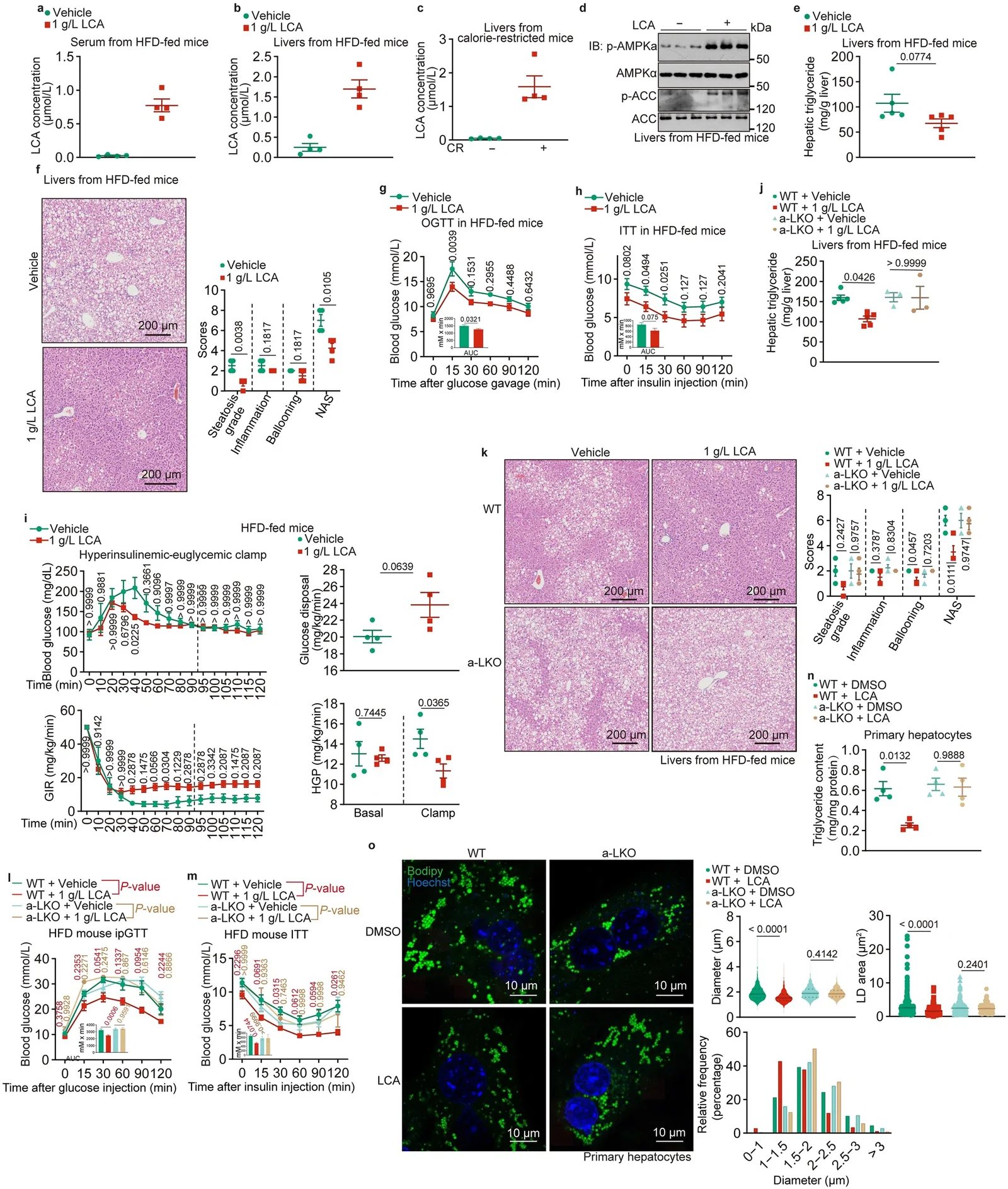
LCA at CR-mimetic doses alleviates high-fat diet-induced fatty liver in mice. (a–c) LCA administration via drinking water (1 g/L) results in exposure levels comparable to those observed in the serum and livers of calorie-restricted mice. Obese mice with fatty liver were used, which were induced by feeding 4-month-old mice with an HFD for 5 months. These obese mice were treated with 2-hydroxypropyl-β-cyclodextrin-coated LCA or the vehicle control for 1 month. The concentrations of LCA in the serum (a) and liver tissues (b) of these mice, and mouse livers after 3.5 months of CR (c) as a control, were measured. Results are mean ± SEM; *n *= 4 mice for each condition. (d) LCA activates AMPK in the livers of the obese mice. Mice were treated as in (b). Liver tissues were excised and lysed, followed by determination of AMPK activation by determining phosphorylated AMPK (p-AMPKα) and phosphorylated ACC (p-ACC) using immunoblotting. (e and f) LCA reduces fatty liver in obese mice. Mice were treated as in (b), followed by determination of hepatic TAG levels (e) and liver section morphology (f; by H&E staining, representative images are shown in the left panel, and the nonalcoholic fatty liver disease [NAFLD] activity score [NAS, including ballooning score, steatosis grade, and the lobular inflammation score] is shown in the right panel). Results in (e) and (f) are mean ± SEM; *n *= 5 (e) or 4 (f) mice for each condition, with *P*-value analyzed using two-sided Student’s *t*-test with Welch’s correction (inflammation and ballooning in (f)) or two-sided Student’s *t*-test (others). (g–i) LCA ameliorates insulin resistance in the obese mice. Mice were treated as in (b), and subjected to OGTT (g). The results for blood glucose levels and the areas under the curve (AUC) during OGTT are shown. ITT was also performed (h), and blood glucose levels and AUC are shown. In addition, the hyperinsulinemic-euglycemic clamp was performed (i). Blood glucose levels and the GIR values during the clamp are shown (i, left panel; *n *= 4 mice for each condition), and the glucose disposal rates and the HGP rates, calculated according to the average GIR values during the steady state (90–120 min, indicated by a dashed line), are shown (i, right panel). Data are shown as mean ± SEM, *n *= 5 (g), *n *= 6 (h), or *n *= 4 (i) mice for each treatment, with *P*-values calculated by two-way repeated-measures (RM) ANOVA followed by Sidak’s test (comparing blood glucose levels of (g) and (h), and GIR values of (i), between the vehicle and LCA groups at each time point), or two-sided Student’s *t*-test (AUC of (g) and (h), and glucose disposal rates and the HGP of (i)). (j–m) LCA alleviates fatty liver and improves insulin sensitivity in obese mice in an AMPK-dependent manner. The *AMPKα*-LKO (α-LKO) mice and WT littermates were fed with HFD for 5 months to induce obesity and fatty liver, followed by LCA treatment as in (b) (see validation data for the knockout efficiency of AMPK in Supplementary Fig. S1c). Hepatic TAG levels (j), liver section morphology (k), NAS (k), and the sensitivity of insulin as assessed by intraperitoneal glucose tolerance test (ipGTT; l) and ITT (m) were determined. Results are mean ± SEM; *n *= 3 (α-LKO of (j)), *n *= 4 (α-LKO of (l) and (m), and (k)), or *n *= 5 (others) mice for each genotype/treatment, with *P*-values calculated by two-way RM ANOVA (blood glucose of (l) and (m)) or two-way ANOVA (others), followed by Tukey. (n and o) LCA reduces lipid accumulation in hepatocytes in an AMPK-dependent manner. Primary hepatocytes isolated from mice were treated with 250 μmol/L OA for 12 h, and then 1 μmol/L LCA for an additional 4 h. The TAG contents (n; mean ± SEM; *n *= 4 dishes of cells for each condition) were determined, and the morphology of the lipid droplets, after staining with BODIPY (4,4-difluoro-4-bora-3a, 4a-diaza-s-indacene) dye (o), was examined. Representative images (o, left panel) and quantification of the total area and size (measured as average diameters and the percentages/distributions of each diameter range) of lipid droplets (right panel, mean ± SEM; *n *= 823–1414 lipid droplets from 8 to 14 cells for each condition) are shown. *P*-values were calculated by two-way ANOVA followed by Tukey’s test.

We also analyzed the LCA-treated mice for any sign of hepatotoxicity, a concern arising from studies using pathological models with excessively high exposures [9–17]. We found that CR-mimetic doses of LCA had little, if any, hepatotoxicity, as serum levels of alanine aminotransferase (ALT; Fig. 2a) and aspartate aminotransferase (AST; Fig. 2b) remained unchanged. Histological examination of liver sections also revealed normal hepatocyte morphology, with no signs of injury, as assessed by H&E staining (Fig. 1f). In addition, there were no significant changes in macrophage or neutrophil infiltration, as shown by immunohistochemistry for F4/80 and Ly6G (Fig. 2c). We also did not see any increase of apoptotic activity according to terminal deoxynucleotidyl transferase dUTP nick-end labeling (TUNEL) staining or cleaved caspase-3 levels, or hepatic fibrosis as evidenced by Sirius Red staining (Fig. 2c). Furthermore, the size of the gallbladder was unaffected (Fig. 2d), indicating the absence of cholestasis. These observations suggest that LCA, at CR-mimetic doses, can alleviate fatty liver without causing hepatotoxicity in mice, even with ­obesity. We also tested the safety of LCA in mice of different ages. In aged (1-year-old) mice, LCA did not show hepatic cytotoxicity (Fig. 2e–i), and even reversed age-related dysfunction in the liver by increasing NAD^+^ levels (Fig. 2j), a key downstream effector of AMPK with anti-aging functions [26]. Moreover, age-related fibrosis (Fig. 2h) and TAG accumulation (Fig. 2k) in the liver were significantly reduced by LCA treatment. To further ascertain the safety of LCA, we also administered CR-mimetic doses of LCA to young mice, and again detected no sign of hepatotoxicity (Supplementary Fig. S3a–f). However, the hepatotoxicity of LCA became apparent at exposure levels much higher than the already high concentration in calorie-restricted mouse serum, as shown at 250 mpk per day via intraperitoneal injection (125 mpk twice a day) for 4 consecutive days to mice, consistent with such high dosage being hazardous to the liver [9–11]. This treatment resulted in LCA accumulation of approximately 14 μmol/L in the liver (more than 10 times higher than that in CR mice, equivalent to over 200 times that in *ad libitum* fed mice; Supplementary Fig. 3a) and 5 μmol/L in the serum (5 times higher than that in CR mice; Supplementary Fig. 3a), and caused significant liver damage (Supplementary Fig. S3c–f). Such extremely high LCA, up to 100 μmol/L in some previous studies [1, 15–17], also caused cell death, observed via Annexin V staining (Supplementary Fig. S4a–d), and DNA damage, assessed by phosphorylated histone H2AX at Ser 139 (γH2AX) foci (Supplementary Fig. S4e–g) in mouse and human hepatocytes. On the contrary, at the CR-mimetic levels of 1 μmol/L, LCA elevated NAD^+^ levels (Supplementary Fig. S4h), indicating that LCA enhanced cellular function and attained rejuvenation. Together, these results suggest that LCA, at CR-mimetic doses, can recapitulate the benefits of CR with no adverse cost.

**Figure 2:**
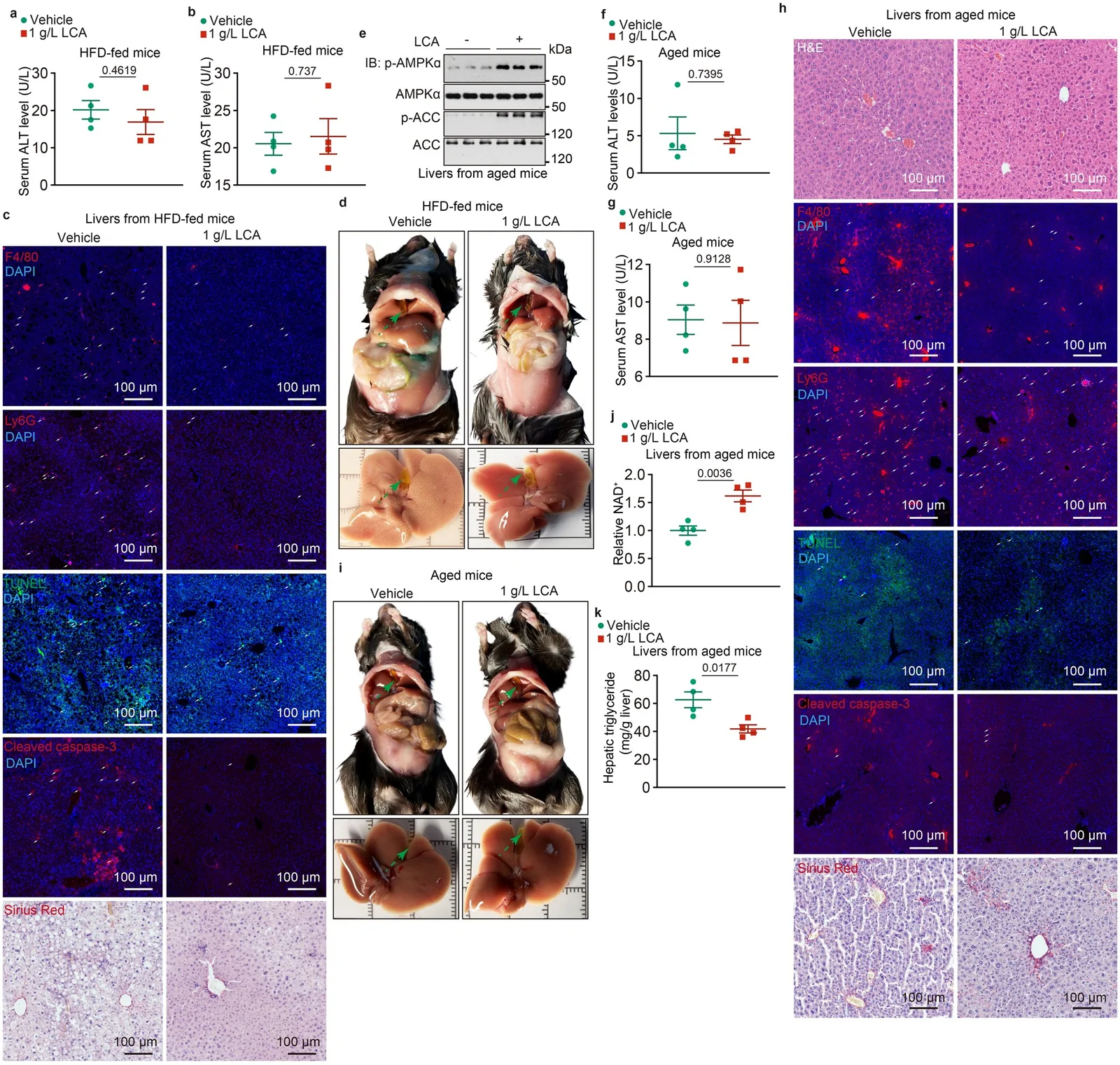
LCA at the high level present in calorie-restricted mice does not cause hepatotoxicity. (a–d) LCA reduces fatty liver in obese mice without causing liver damage. Mice were fed with HFD, followed by administration of the CR-mimetic dose of LCA for 1 month, as in Fig. 1e. After treatment, serum and liver samples from these mice were collected. The serum levels of ALT and AST (a and b; data are mean ± SEM; *n *= 4 mice for each condition, with *P*-value calculated using two-sided Student’s *t*-test), the macrophage (c, “F4/80”) and neutrophil (c, “Ly6G”) infiltration, the presence of apoptotic hepatocytes (c, “TUNEL” and “Cleaved caspase-3”), and the fibrosis (c, “Sirius Red”) of liver section were assessed. Cells with positive staining for each marker are indicated by white arrows. See also Fig. 1f for the morphology (“H&E” panels) of the liver section. Representative images of the liver are also shown (d), with the gallbladder indicated by green arrows. (e–k) LCA alleviates age-related fatty liver without causing damage. Aged mice were administered the CR-mimetic dose of LCA for 1 month, a treatment previously shown to effectively rejuvenate muscles [1, 2]. Following the treatment, serum and liver samples were collected. The activation of AMPK in the liver (e), along with the levels of NAD^+^ (j, mean ± SEM; *n *= 4 mice for each condition) and TAG (k, mean ± SEM; *n *= 4 mice for each condition) levels were measured. Liver damage indices, including ALT and AST levels in the serum (f and g; data are mean ± SEM; *n *= 4 mice for each condition), were also analyzed. In addition, the liver sections were examined for morphology (h, “H&E”), fibrosis (h, “Sirius Red”), macrophage (h, “F4/80”) and neutrophil (h, “Ly6G”) infiltration, and the presence of apoptotic hepatocytes (h, “TUNEL” and “Cleaved caspase-3”). Cells with positive staining for each marker are indicated by white arrows. Representative images of the liver are shown in (i), with the gallbladder indicated by green arrows. *P*-values in these panels are calculated by two-sided Student’s *t*-test.

We next determined the effects of LCA on fatty liver as well as its dose-dependent safety in macaques. We enrolled a cohort of 21 monkeys (Supplementary Table S1), and induced obesity and fatty liver in them by feeding with HFD for 8 months. All the monkeys turned obese, and 17 of them developed fatty liver as assessed by histopathology (liver biopsy) and ultrasonography; these 17 monkeys with fatty liver were subjected to further expe­riments (Supplementary Table S2). Of note, these monkeys did not show elevated fasting blood glucose levels, glycated hemoglobin A1c (HbA1c), or serum TAG, although total cholesterol (TC) and low-density lipoprotein cholesterol (LDL-C) were higher than the reference values for healthy cynomolgus macaques (Supplementary Table S2).

We first administered LCA via drinking water at 1 g/L, as in mice, using the same 2-hydroxypropyl-β-cyclodextrin formulation. However, within 1 week of administration, the monkeys experienced a sharp decline in water intake (Fig. 3a), possibly due to a gustatory reason or taste aversion. Adding 10% glucose to mask the taste failed to restore normal drinking (Fig. 3a). This could potentially cause a risk of dehydration, which is of animal welfare concerns. We therefore had to develop an alternative formulation of administration. After screening several candidates, we coated LCA with phospholipids and dissolved it in fish oil (see Methods section for details), which helped mitigate the taste aversion in monkeys. In mice, we confirmed that gavaging this new formulation at 30 mpk resulted in the same CR-mimetic serum LCA concentration (approximately 1 μmol/L; Fig. 3b; with a half-life of approximately 1 h), close to the concentrations achieved following CR in mice and humans [1, 27, 28]. Based on body surface area conversion [29], we calculated a corresponding dose of 9.6 mpk for monkeys. Unexpectedly, we found that this dose rapidly reached a peak serum LCA concentration in monkeys exceeding 6 μmol/L just 30 min after gavage (Fig. 3c), approximately 50 times higher than the levels observed in ungavaged monkeys or humans. Such high levels of LCA caused hepatotoxicity within a week, as evidenced by significant elevations in serum ALT and AST levels (Fig. 3d).

**Figure 3:**
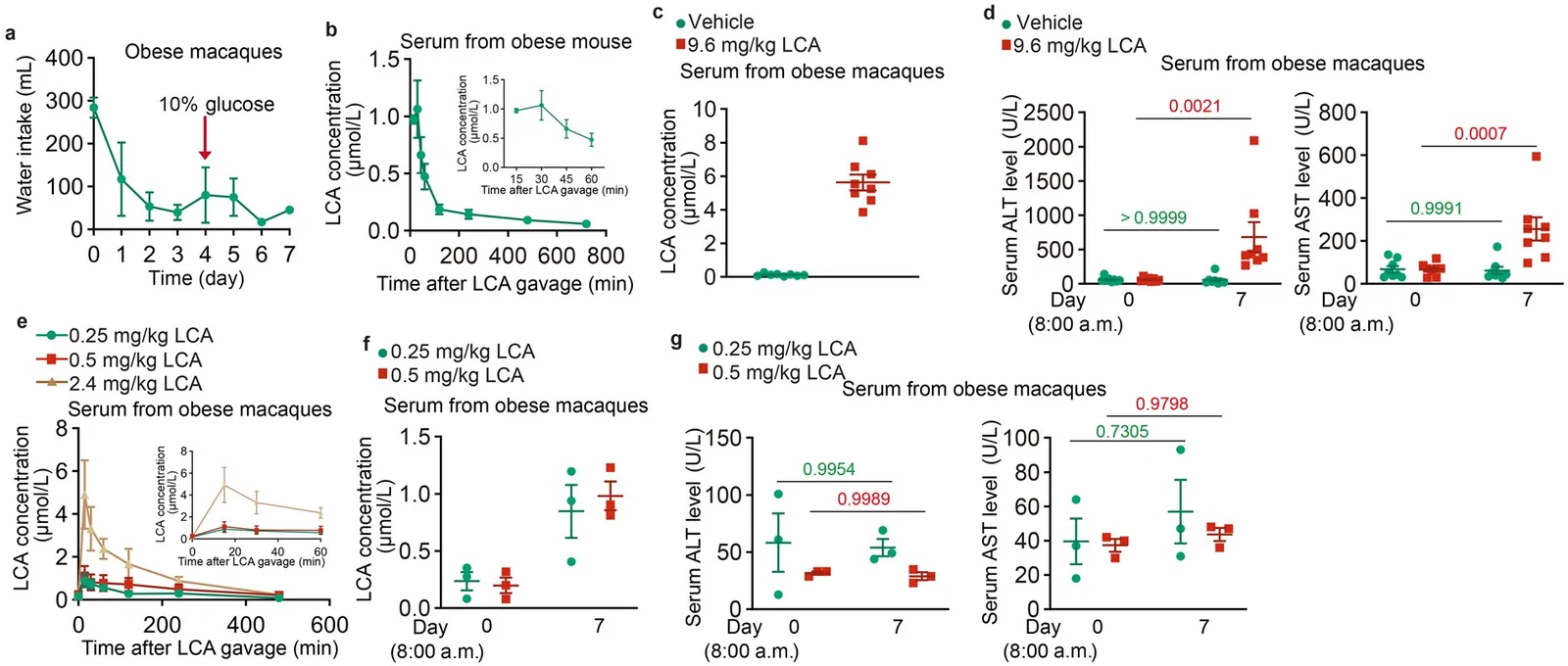
Titration of LCA doses for administration to macaques. (a) Administration of LCA via the same route as that used in mice leads to a rapid decrease in water intake in monkeys. Monkeys were fed with an HFD to induce fatty liver and obesity. Initially, monkeys were administrated with 2-hydroxypropyl-β-cyclodextrin-coated LCA in drinking water (1 g/L). After 4 days, 10% glucose was added in LCA-containing drinking water for enhancement of palatability (indicated by the dashed line). This administration led to a decrease in water intake, as measured at the indicated time points after LCA administration. Data are mean ± SEM; *n* = 3 monkeys for each time point. (b–d) The doses of LCA administered to the macaques do not obey the surface area-based conversion from mice to macaques. Obese mice (induced as in Fig. 1a) were gavaged with 30 mpk LCA (coated with phospholipids and dissolved in fish oil), followed by determination of LCA concentrations in the serum at different time points (b). In parallel, obese monkeys (induced as in (a)) were gavaged 9.6 mpk of LCA, the dose calculated through surface area conversions, followed by determination of LCA concentrations in the serum at 30 min after the gavage (c), when LCA reached peak concentrations in the serum of mice. In addition, liver enzymes ALT and AST in the serum of these monkeys were measured after twice-daily gavage for 7 consecutive days (d). Data are mean ± SEM, *n* = 4 mice (b) or 8 monkeys (c and d) for each time point/condition, with *P*-values in d calculated using two-way ANOVA followed by Tukey’s test. (e–g) Titration of LCA doses for administration to macaques. Obese monkeys were gavaged with various doses of LCA: 2.4 mpk (e), 0.5 mpk (e–g), and 0.25 mpk (e–g), either once (e) or twice daily for 7 consecutive days (others), to determine a specific dosage that resulted in blood exposure comparable to that observed in the serum of calorie-restricted monkeys and humans. Blood samples were collected at the indicated time points after acute LCA administration (e) or at 08:00 a.m. on specified days after consecutive dosing (f and g). The levels of LCA (e and f), ALT, and AST (g) in the serum were measured. Results are mean ± SEM; *n* = 3 monkeys for each condition, with *P*-values calculated using two-way ANOVA followed by Tukey’s test.

This peculiarly high exposure, severely deviating from the expected values, prompted us to perform dose titration in monkeys. As shown in Fig. 3e, we found that LCA at doses of 0.25 mpk and 0.5 mpk achieved peak serum concentrations of approximately 0.9 and 1.1 μmol/L at 15 min post-gavage, respectively (both with a half-life of approximately 4 h), whereas a dose of 2.4 mpk resulted in approximately 5 μmol/L. We thus selected 0.25 and 0.5 mpk for further experiments. Twice-daily gavage at these doses for 1 week yielded steady-state serum LCA concentrations of approximately 0.8 μmol/L and 1 μmol/L, respectively (Fig. 3f). Hepatic toxicity analysis showed no elevation of ALT and AST levels in the serum, indicating that such serum exposure levels did not cause hepatotoxicity (Fig. 3g).

We next divided these 17 monkeys into three groups, receiving either vehicle, 0.25 mpk LCA, or 0.5 mpk LCA, by twice-daily gavage for 13 weeks. Throughout this period, we monitored food intake, body weight, and blood biochemistry weekly; prior to and at the end of the LCA administration, we performed body composition analysis, liver ultrasound, and liver biopsy histopathology (depicted in Fig. 4a). After 13 weeks of treatment, we observed a marked reduction in fatty liver in both LCA-treated groups compared to vehicle as assessed by H&E staining (Fig. 4b; Supplementary Fig. S5), which was corroborated by results from quantitative ultrasound analysis (Supplementary Fig. S6). Body weight and body fat percentage remained unchanged over the 13-week period in all groups, nor did we observe significant alterations in serum total cholesterol, TAG, LDL, high-density lipoprotein (HDL), fasting glucose, or HbA1c (Fig. 4c–j; with other physiological indexes unchanged, see Supplementary Table S2). Importantly, LCA treatment at both doses did not cause any detectable liver toxicity in the monkeys, as assessed by serum ALT and AST levels (Fig. 4k). Additionally, renal function, as indicated by the levels of serum creatinine, was unaffected (Fig. 4l), and no adverse changes were noted in blood cell counts and percentages (Supplementary Table S2). These findings demonstrate that administration of LCA at CR-mimetic doses also alleviates fatty liver in non-human primates, without causing any toxicity.

**Figure 4:**
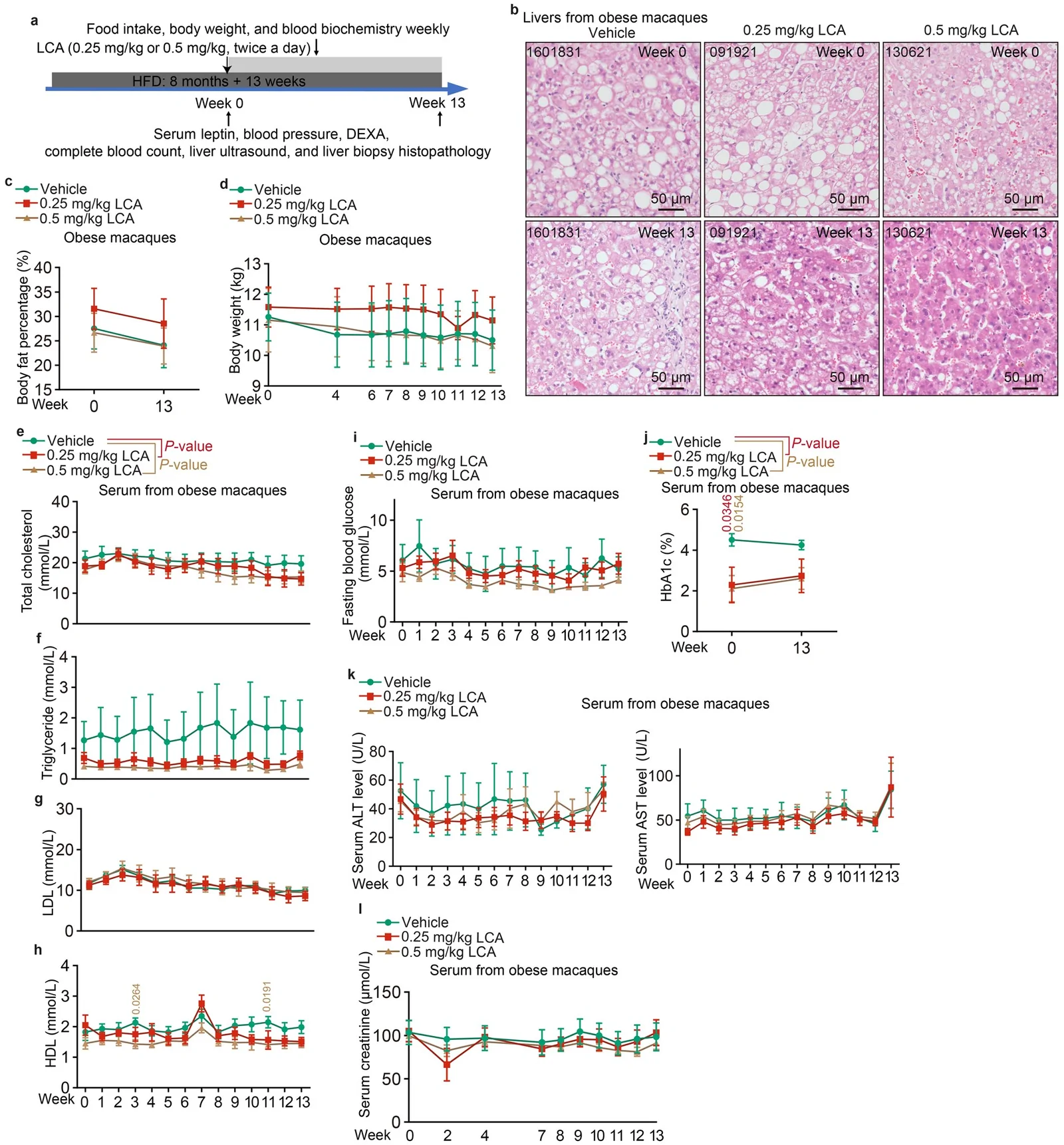
LCA alleviates fatty liver in macaques. (a) A schematic diagram illustrating the timeline and procedures of the experiments to explore the role of LCA in alleviation of fatty liver in macaques. (b–l) LCA alleviates fatty liver in macaques. Monkeys were fed an HFD, as in Fig. 3a. A total of 17 (out of 21) monkeys that developed fatty liver were gavaged with LCA at 0.25 mpk and 0.5 mpk, twice daily for 13 weeks. The following measurements were performed at indicated time points after LCA administration: morphology of liver sections (b; representative images for each dose are shown, with the identification numbers of the monkeys labeled; additional images for other individual monkeys can be found in Supplementary Fig. S5), body fat percentage (c, assessed by dual-energy X-ray absorptiometry [DEXA]), body weight (d), levels of total cholesterol (e, determined after a 15-h fast, and the same for the rest of the panels), TAG (f), LDL (g), and HDL (h), as well as fasting blood glucose (i) and HbA1c (j) levels. Toxicity indices, including serum ALT and AST levels (k) and serum creatinine (l), were also assessed. Results are mean ± SEM; *n* = 5 (0.25 mpk) or *n* = 6 (others) macaques for each treatment, with *P*-values calculated by two-way ANOVA, followed by Tukey, comparing the vehicle group with the 0.25 mpk LCA group (indicated by red numbers) and the vehicle group with the 0.5 mpk LCA group (indicated by brown numbers) at each time point.

We also found that alleviation of fatty liver by LCA is virtually blocked when AMPK is knocked out in the liver, in line with a general role of AMPK in reducing fat content in the liver [30, 31]. In addition, we found that LCA plays an augmentative role in GLP-1 secretion, consistent with its ability to also bind to bile acid receptors, farnesoid X receptor (FXR) [32, 33] and Takeda G protein-coupled receptor 5 (TGR5) [34], both of which are involved in GLP-1 secretion [35, 36] and can alleviate fatty liver [37–39]. GLP-1 can also activate AMPK in the liver via the GLP-1 receptor (GLP-1R)-cAMP-Ca^2+^ cascade [40]. Therefore, LCA with GLP-1 or GLP-1 receptor agonists (GLP-1RAs) may converge on hepatic AMPK activation to alleviate fatty liver. It is also noteworthy that activation of thyroid hormone receptor-β (THR-β) has been reported to stimulate β-oxidation of fatty acids in the liver to alleviate fatty liver [41]. Therefore, it is possible that combinational therapies yield better benefits in treating fatty liver patients.

We have also shown that LCA, at CR-mimetic concentrations, does not cause liver or organ toxicity in either mice or macaques. LCA causes hepatotoxicity only when exceeding 5 times higher than the already high concentrations in the serum of calorie-restricted mice or humans. It is also noteworthy that there are significant differences between species in the pharmacokinetics of LCA. We found that the dose conversion between mice and non-human primates does not conform to standard body surface area-based calculations. Although the exact reason for this discrepancy remains unclear, it stems at least in part from differences in bile acid composition and the metabolic rates observed between rodents and primates [24]. In particular, in mice, LCA can be metabolized in the liver by the cytochrome P450 enzymes CYP2C70 and CYP2A12, producing muricholic acid (MCA) [42, 43]. This conversion does not occur in primates, which may contribute to the significant accumulation of LCA in them. Together, at CR-mimetic doses, LCA is safe and effective as a CR-mimetic agent for non-human primates.

Our study has several limitations. First, the cynomolgus macaques fed with HFD in our study reflect an early stage of metabolic dysfunction with significant hepatic steatosis and elevated body mass index (BMI), but without pronounced hypertriglyceridemia or hyperglycemia [44]. Therefore, it is possible that LCA treatment could also improve these parameters in more advanced metabolic disease models. Additionally, although our data establish a dose-exposure relationship in monkeys, we did not fully characterize the pharmacokinetic profiles, including tissue distribution and potential sex differences. Longer treatment durations, broader metabolic disease models, and pharmacokinetics assessment will be necessary to fully understand the therapeutic potential of LCA in primates, including humans.

## Supplementary Material

loag023_Supplementary_Data

## Data Availability

The analysis was performed using standard protocols with previously described analysis tools. Materials and reagents are available upon request. Questions regarding the details of experiments are welcome.

## Code availability

No custom code was used in this study.
