# Supplementary material for: Lithocholic acid alleviates fatty liver in mice and non-human primate macaques
Source: Life Metab. 2026 Jul 23;5(6):loag023. doi: 10.1093/lifemeta/loag023 (PMC13591641; doi:10.1093/lifemeta/loag023)
Supplement: Supplementary file 1 [file loag023_supplementary_data.zip › LCA_in_monkeys_methods_0715_for_publication.docx]

**Supplementary Information for**

**Lithocholic acid alleviates fatty liver in mice and non-human primate macaques**

Qi Qu^1,2,‡^, Yan Chen^2,‡^, Guo Shen^3^, Xiaojiao Huang^3^, Fang-Han Li^2^, Xiaojun Wang^2^, Cixiong Zhang^2^, Zhumei Wei^3^, Liping Xie^2^, Jianfeng Wu^4^, Jinlong Yang^5^, Qianru Xu^1^, Nan Xu^1^, Weiqi Xu^6^, Su Chen^1^, Jianping Qi^5^, Chen-Song Zhang^1,2,7^, Sheng-Cai Lin^1,2,7,*^

^1^Zhongzhou Laboratory for Integrative Biology, School of Basic Medical Sciences, Henan University, Kaifeng, Henan 475004, China

^2^State Key Laboratory of Cellular Stress Biology, School of Life Sciences, Faculty of Medicine and Life Sciences, Xiamen University, Xiamen, Fujian 361102, China

^3^Guangxi Huaren Jiying Biotechnology Co., Ltd., Nanning, Guangxi 530000, China

^4^Laboratory Animal Research Center, Xiamen University, Xiamen, Fujian 361102, China

^5^School of Pharmaceutical Sciences, Key Laboratory of Smart Drug Delivery, State Key Laboratory of Advanced Drug Formulations, Fudan University, Shanghai 201203, China

^6^Department of Hepatic Surgery, Shanghai Cancer Center; Department of Oncology, Shanghai Medical College, Fudan University, Shanghai 200032, China

^7^Department of Gastrointestinal Surgery, Institute of Gastrointestinal Oncology, Zhongshan Hospital of Xiamen University, Xiamen, Fujian, 361004, China

^‡^These authors contributed equally

^*^Corresponding author. Zhongzhou Laboratory for Integrative Biology, School of Basic Medical Sciences, Henan University, Kaifeng, Henan 475004, China. E-mail: [linsc@xmu.edu.cn](mailto:linsc@xmu.edu.cn)

**Supplementary Figure** **S1** LCA alleviates high-fat diet-induced fatty liver in mice.

(a and b) LCA reduces fat content in HFD-fed mice. Mice were fed with an HFD to induce fatty liver, and then treated with 2-hydroxypropyl-β-cyclodextrin-coated LCA at 1 g/L in drinking water, which resulted in exposure levels similar to those found in the serum and livers of calorie-restricted mice (as shown in Fig. 1a−c), over a period of 1 month. During the treatment, the body weights of the mice were measured every other day (b). At the end of the treatment, body weights, as well as the weights of white adipose tissues (WATs; the sum of inguinal, perirenal, gonadal, and mesenteric white adipose tissue weights), liver, brown adipose tissue (BAT), and gastrocnemius (Gas) were determined (a). Additionally, tissue/organ-to-body weight ratios were calculated (a). Data are mean ± SEM; *n* = 4 (b, and the weights of gastrocnemius in the LCA-treated group in a) or *n* = 5 (a) mice for each condition, with *P*-values calculated by two-sided Student’s *t*-test (a), or by two-way ANOVA, followed by Sidak (b). (c) Validation of AMPKα knockout efficiency in the livers of *AMPKα*-LKO mice. The *AMPKα*-LKO (α-LKO) mice and wild-type (WT) littermates, both fed with an HFD for 5 months, were treated with LCA as in Fig. 1k. The expression of AMPKα in liver tissues, along with the activation of AMPK, was determined by immunoblotting analysis. (d) LCA promotes postprandial GLP-1 secretion. WT mice were fed with an HFD for 5 months to induce fatty liver, and were treated with LCA as in Fig. 1b for 2 week. Mice were then fasted for 16 h, followed by gavaging with 2 g/L glucose, and then sacrificed at the indicated time intervals following the glucose gavage. Levels of GLP-1 (total levels, including full-length and two truncated forms) in the plasma were measured. Results are shown as mean ± SEM; *n* = 4 mice for each time point, and *P*-values by two-way ANOVA, followed by Tukey, all compared to the fasting group.

**Supplementary Figure** **S2** LCA decreases lipid accumulation in hepatocytes.

(a−d) LCA activates AMPK in cultured hepatocytes. The primary-like human HepaRG hepatocytes (a and b) and human hepatoma HepG2 cells (c and d) were treated with 1 µmol/L LCA in the culture media for 4 h, followed by determination of intracellular concentrations of LCA (a and c; data are mean ± SEM; *n* = 4 dishes of cells for each treatment) and AMPK activity (b and d). (e−h) LCA decreases lipid accumulation in hepatocytes. HepaRG cells (e and f) and HepG2 cells (g and h) were treated with 250 μmol/L oleic acid (OA) for 12 h, followed by incubation with 1 μmol/L LCA for an additional 4 h. The triglyceride contents (f and h; results are mean ± SEM; *n* = 4 dishes of cells for each condition, with *P*-value calculated by two-sided Student’s *t*-test) were determined, and the morphology of the lipid droplets, after staining with BODIPY dye (e and g), was examined. In panels e and g, representative images (left panels) and quantification of the total area and size (measured as average diameters and the percentages/distributions of each diameter range) of lipid droplets (right panels, results are mean ± SEM; *n* = 1128−1306 lipid droplets from 18−25 cells for each condition, with *P*-values analyzed using two-sided Student’s *t*-test with Welch’s correction (average diameters and area in e) or two-sided Student’s *t*-test (others)) are shown.

**Supplementary Figure** **S3** LCA at CR-mimetic doses does not cause liver damage.

Lean mice of 4 months old were administered the CR-mimetic dose, 1 g/L LCA in drinking water for 1 month, or received intraperitoneal injections of 250 mpk LCA for 4 consecutive days as a positive control to induce liver damage. Following these treatments, serum and liver samples were collected for analysis. The concentrations of LCA in the serum and livers (a), and the activation of AMPK (b) in the livers were determined. Liver damage indices, including serum ALT and AST levels (c and d), were determined. In addition, liver sections were analyzed for morphology (e, “H&E”), fibrosis (e, “Sirius Red”), macrophage (e, “F4/80”), and neutrophil (e, “Ly6G”) infiltration, and the presence of apoptotic hepatocytes (e, “TUNEL” and “Cleaved caspase-3”). Cells with positive staining for each marker are indicated by white arrows. Representative images of the liver are shown in panel f, with the gallbladder indicated by green arrows. Results are mean ± SEM; *n* = 4 mice for each condition, with *P*-values calculated by two-way ANOVA, followed by Tukey.

**Supplementary Figure** **S4** LCA at CR-mimetic dose does not cause cell death or DNA damage in hepatocytes.

(a−d) LCA does not cause cell death in cultured hepatocytes. HepaRG (a and b) and HepG2 (c and d) cells were treated with the CR-mimetic dose, 1 μmol/L LCA, or 100 μmol/L LCA as a positive control to induce cell death, for 12 h. Cell death was assessed using Annexin V-PI staining followed by flow cytometry. During the analysis, intact cells from each sample were selected by FSC-H and SSC-H (left panels of a and c, using a linear scale), followed by FSC-H and FSC-A to exclude doublets (middle panels of a and c, using a linear scale). The fluorescence intensities of propidium iodide (PI) and Annexin V-FITC were then determined and presented as density plots (right panels of a and c, using a logarithmic scale). The plot is divided into four quadrants (Q1, Q2, Q3, and Q4), in which Q1 (Annexin V-negative and PI-positive populations) represents necroptotic cells, Q2 (Annexin V-positive and PI-positive populations) represents late apoptotic cells, and Q3 (Annexin V-positive and PI-negative populations) represents early apoptotic cells. The percentages of early apoptotic, late apoptotic, and necroptotic cells are shown at the bottom of panels of b and d as means ± SEM; *n* = 4 dishes of cells, with *P*-values calculated by two-way ANOVA, followed by Tukey. See also representative density plots on the top of panels of b and d. (e−g) CR-mimetic LCA does not cause DNA damage in hepatocytes. HepaRG cells (e), HepG2 cells (f), and primary hepatocytes (g) were treated with 1 μmol/L or 100 μmol/L LCA for 12 h, or exposed to 75 J/m^2^ UV followed with incubation in fresh DMEM for another 2 h as a control for DNA damage. Cells were then lysed, followed by determination of the levels of H2AX and RPA32 phosphorylation by immunoblotting. (h) LCA elevates NAD^+^ levels in hepatocytes. Primary hepatocytes were treated with 1 μmol/L LCA for 24 h, followed by determination of NAD^+^ levels. Results are mean ± SEM; *n* = 4 dishes of cells for each condition, with *P*-values calculated by two-sided Student’s *t*-test.

**Supplementary Figure S5** LCA reduces fatty liver in macaques.

Monkeys were fed an HFD to induce fatty liver, followed by LCA (coated with phospholipids and dissolved in fish oil) gavage at doses of 0.25 mpk (middle panels) and 0.5 mpk (right panels), or vehicle (left panels), twice a day for 13 weeks. Representative images of liver sections for each individual monkey (with the identification numbers of the monkeys labeled) before and after the 13-week LCA administration are shown.

**Supplementary Figure** **S6** LCA decreases liver echogenicity in macaques.

Monkeys were fed an HFD to induce fatty liver, followed by LCA gavaging for 13 weeks (as in Fig. 4a). Liver ultrasound images were taken for assessing the severity of fatty liver. Representative images for each individual monkey before and after the 13-week LCA administration are shown. In each image, red arrows point to the near field (the region relatively close to the probe marker) and the green arrow indicates the far field (the region relatively distant from the probe marker). The degree of posterior acoustic attenuation was then evaluated by comparing the brightness between these two fields. If the far field appears darker than the near field and a larger darkened area is observed, the hepatic steatosis is more severe. Additionally, the yellow line segment connects the liver (at one end) to the renal cortex (at the other end; which provides an internal reference for echogenicity). A liver echogenicity lower than that of the renal cortex suggests a healthy liver, whereas an echogenicity that is equal to or greater than that of the renal cortex indicates fatty liver. See also Supplementary Table S4 for the severity grades evaluated based on the ultrasound images of each monkey.

**Supplementary Table S1** Baseline physiological indexes for each of the 21 monkeys enrolled for obesity induction.

**Supplementary Table S2** Physiological indexes for each of the 17 obese monkeys before and after 13 weeks of LCA administration.

**Supplementary Table S3** Average food and calorie intake for each of the 17 obese monkeys during each week of the LCA administration.

**Supplementary Table S4** Summary of the diagnosis and grading of fatty liver for each obese monkey.

1	Qu, Q. *et al.* *Nature* (2024).

2	Qu, Q. *et al.* *Nature* (2024).

**Materials and methods**

**Data reporting**

The chosen sample sizes were consistent with those commonly used in this field. Specifically, the following sample sizes were utilized: *n* = 3−8 samples to assess the levels of lithocholic acid (LCA) in serum^1-3^, cells^3,4^, and tissues^3,5^; *n* = 3−6 mice^6,7^ and monkeys^8-11^ to determine the blood chemistry; *n* = 3−6 mice to evaluate liver fibrosis, hepatocyte injury, and hepatocyte apoptosis^12-14^; *n* = 3−6 mice to determine immune cell infiltration in liver tissues^7,13,15^; *n* = 4 liver tissues to measure the levels of NAD^+^ (ref. ^16,17^); *n* = 3−6 mice^6,18,19^ and monkeys^10,20,21^ to evaluate the hepatic steatosis and triglyceride (TAG) levels; *n* = 2−3 cell and liver samples to determine the expression and phosphorylation levels of AMPK and ACC^22-24^; *n* = 4 cell samples to determine the levels of apoptotic markers^7,25-27^; *n* = 5−6 mice^3,6^ and monkeys^28,29^ for body composition and fat mass measurements; and *n* = 4−5 mice for performing oral glucose tolerance test (oGTT), insulin tolerance test (ITT), and hyperinsulinemic-euglycemic clamping^3,6^. No statistical methods were used to pre-determine the sample size. All experimental findings were repeated as stated in the figure legends, and all additional replication attempts were successful. For animal experiments, mice and monkeys were housed under the same conditions or in the same places. For cell experiments, cells of each genotype were cultured in the same CO_2_ incubator and were seeded in parallel. Each experiment was designed and performed along with controls, and samples for comparison were collected and analyzed under the same conditions. Randomization was applied wherever possible. For example, during mass spectrometry (MS) analyses of NAD^+^ and LCA levels, samples were processed and subjected to the MS in random orders. For animal experiments, age-matched littermates in each genotype were randomly assigned to LCA or vehicle treatment groups. In cell experiments, cells of each genotype were parallel-seeded and randomly assigned to different treatments. Otherwise, randomization was not performed. For example, when performing immunoblotting, samples needed to be loaded in a specific order to generate the final figures. Blinding was applied wherever possible. For example, samples or cages during sample collection and processing were labeled with code names that were later revealed by the individual who picked and treated animals or cells, but did not participate in sample collection and processing, until the outcome was assessed. Similarly, during microscopy data collection and statistical analyses of lipid droplets in hepatocytes, fields of view were chosen at random, and were performed by different operators, preventing potentially biased selection of desired phenotypes. Otherwise, blinding was not performed. For example, for assessing the toxicity of LCA in monkeys, the operators needed to know the group allocations. This was essential for monitoring the monkeys’ behavior and health in real time, allowing for emergency procedures and interventions and for enforcing the humane endpoint in accordance with ethical standards.

**Mouse use and care**

Protocols for all mouse experiments were approved by the Institutional Animal Care and the Use Committee (IACUC) of Xiamen University (XMULAC20220050). Unless stated otherwise, mice were housed with free access to water and standard diet (65% carbohydrate, 11% fat, and 24% protein) under specific pathogen-free conditions. The light was on from 8:00 to 20:00, with the temperature kept at 21-24 °C and humidity at 40-70%. Male littermate controls were used throughout the study.

For generating the obese mice with fatty liver, mice were fed an HFD (60% calories from fat; D12492, Research Diets) for 5 months starting at 4 months old. Caloric restriction (CR) was performed as described previously^3,30^. Briefly, mice were individually caged for 1 week before the experiment. Each mouse was fed 2.5 g of standard diet (approximately 70% of *ad libitum* food intake for a mouse at 4 months old and older) at 17:00 each day.

The mouse strains used in this study were obtained or generated as follows. Wild-type (WT) C57BL/6J mice (#000664) were obtained from The Jackson Laboratory. *AMPKα1^F/F^* (#014141) and *AMPKα2^F/F^* mice (#014142) were obtained from Jackson Laboratory, provided by Dr. Sean Morrison. The inducible liver-specific AMPKα knockout (*AMPKα*-*LKO*) mice were generated by crossing *AMPKα1*/2^F/F^ mice with *Alb*-*CreERT2* mice, as described previously^6^. To delete hepatic *AMPKα1* and *AMPKα2*, tamoxifen (dissolved in corn oil) was administered via intraperitoneal injection at a dosage of 200 mg/kg, three times a week. The knockout efficiencies were analyzed 1 week after the final injection using immunoblotting. All mouse strains used in this study were on the C57BL/6J background.

In this study, mice of different ages were used for the following analyses: (a) For analyzing AMPK activation and liver sectioning, WT and *AMPKα*-LKO HFD-fed mice of 10 months old, and WT mice on a normal chow diet of 12 months old, were used. (b) For measuring body weight and body composition, WT HFD-fed mice of 10 months old were used. (c) To measure hepatic TAG, WT and *AMPKα*-LKO HFD-fed mice of 10 months old, and WT mice on a normal chow diet aged 12 months, were used. (d) For the ipGTTs and ITTs, WT and *AMPKα*-LKO HFD-fed mice of 10 months old were used. (e) In the oGTTs, ITTs, and hyperinsulinaemic-euglycaemic clamp analyses, WT HFD-fed mice of 10 months old were used. (f) For determining the concentrations of LCA in serum and liver tissues, WT mice on a normal chow diet of 4 weeks old (only for single dose of administration) or 4 months old (for chronic dosages), WT HFD-fed mice of 10 months old, and WT calorie-restricted mice of 7.5 months old were used. (g) For measuring NAD^+^ levels, WT mice on a normal chow diet of 12 months old were used. (h) To determine the hepatotoxicity of LCA, WT HFD-fed mice of 10 months old and WT chow diet-fed mice of 12 months and 4 months old were used.

The following experiments and measurements were conducted using the same batch of mice: (a) Evaluation of body weight and body composition, measurement of serum ALT and AST levels and hepatic TAG and NAD^+^ levels, imaging of the mouse liver and gallbladder, and histological examination of liver sections for assessing markers of liver damage (morphological analysis, fibrosis assessment, and immunohistochemistry analyses of TUNEL-positive liver cells, F4/80-positive cells, Ly6G-positive cells, and cleaved caspase-3-positive cells); (b) Assessment of liver AMPK activation; (c) Measurement of LCA contents; (d) Performance of hyperinsulinaemic-euglycaemic clamp analyses; (e) Performance of ipGTTs; (f) Conducting ITTs; and (g) Performance of oGTTs.

**Monkey use and care**

***Animal welfare and veterinary care***

All protocols and experimental procedures involving macaques were reviewed and approved by the IACUC of Guangxi Huaren Jiying Biotechnology Co., Ltd. (HRACI-202311-002), which complies with the Chinese National Standard Guidelines for the Ethical Review of Laboratory Animal Welfare (GB/T 35892 2018) as well as the Guide for the Care and Use of Laboratory Animals (National Research Council, 8th edition, 2011). Throughout the study, animal health and welfare were monitored daily by trained animal care staff and a licensed veterinarian. A standardized clinical observation protocol was used to assess each animal based on the following categories:

(a) Behavior: activity level (overactivity, somnolence, languid, spirit dispirited, sluggish); posture (muscle tension, tremor, rolling, anorthosis); temperament (amorphous type, jitters, aggressivity, excitement); vocalization (tone change, noiselessly); ingestion (appetite drops, vomiting, incontinence).

(b) Appearance: general appearance (piloerection, hair breakage, dry, emaciation, wound, anal prolapse); skin and mucosa (stimulation, impairment, color change, rash, abscess, fester); eyes (tearing, secretions, corneal opacity, scleral congestion, visual field injury, pupillary light response, abnormal pupil size); nose (rhinorrhea, nasosinusitis).

(c) Physiological functions: respiration (rate increase or decrease, anhelation, tussis); salivation (timing relative to dosing, volume, interval, duration); urination (frequency, oliguria, anuria, hematuria); defecation (loose stools, bloody diarrhea, astriction, watery stool).

(d) Dosing site: redness/swelling, induration, fester, blister.

(e) Other unusual conditions: any abnormalities not covered above were recorded in remarks.

Any animal showing signs of distress, illness, or injury received prompt veterinary assessment. A pre-established treatment protocol for common diseases was available, and any pharmacological intervention required prior approval from the IACUC.

***Monkey enrollment and* *fatty liver induction***

Monkeys were reared in natural family groups at Guangxi Xiongsen Primate Experimental Animal Breeding and Development Co., Ltd. (Production License No. SCXK Gui 2021-0004). For the induction of fatty liver, monkeys aged 7 years and older had to meet at least one of the following criteria for enrolment, which render a higher susceptibility to developing obesity:

(a) A most recent serum total cholesterol level higher than 3 mmol/L or TAGs higher than 0.94 mmol/L;

(b) A most recent systolic blood pressure higher than 120 mmHg or diastolic blood pressure higher than 90 mmHg;

(c) An atherosclerosis index higher than 2.5;

(d) A body mass index (BMI) higher than 35 kg/m² or a body condition score (BCS) higher than 3.5;

(e) Fasting blood glucose higher than 7 mmol/L.

In total, 21 adult male monkeys, aged 7−17 years and weighing between 6 and 12 kg, who met these criteria, were enrolled to induce fatty liver. The selected animals were transferred to Guangxi Huaren Jiying Biotechnology Co., Ltd., a facility accredited for non-human primate research (Laboratory Animal Use Permit No. SYXK Gui 2024-0002). Upon arrival, the animals underwent a 14-day quarantine and acclimation period, during which a veterinarian conducted daily clinical observations and comprehensive health assessments, including body weight measurements, serum biochemistry, hematology, and urinalysis. Pathogen screening for *Mycobacterium* *tuberculosis*, *Shigella*, *Salmonella*, helminths, ectoparasites, and *Entamoeba* *histolytica* gave negative results for all animals, and physical examination findings were within normal limits. Baseline serum biochemistry and other individual data for each of the 21 monkeys are provided in Supplementary Table S1.

After the quarantine period, monkeys were transferred to individual stainless-steel cages that meet EU standards for non-human primates. These cages allowed for visual and auditory contact, as well as limited olfactory interaction, with other animals in the same room. The animal room was maintained at a temperature of 16−28°C and a relative humidity of 30%−70%, with a 12-h light/12-h dark cycle (lights on at 08:00). Environmental conditions were controlled by an air conditioning unit from Guangzhou Olanda Energy Saving Technology. Cages were flushed, and the floors were wiped twice daily (in the morning and afternoon). The animal room was cleaned and disinfected weekly. During disinfection, three disinfectants were rotated weekly: a 4% (v/v) solution of 84 disinfectant (containing 5% sodium hypochlorite in the stock solution), a 2% (v/v) solution of benzalkonium bromide, and a 0.1% (v/v) solution of Bestaquam. In addition, a 0.5% (v/v) peracetic acid solution was used for room spray disinfection, and 75% (v/v) alcohol was used for instrument cleaning. For environmental enrichment, cages were furnished with mirrors, colored balls, and soft background music, which was rotated weekly. Additionally, daily direct interactions between humans and monkeys were implemented twice a day, and peanuts and sunflower seeds were provided to help reduce stress when humans were present. All enrichment procedures, including foraging opportunities and positive reinforcement training, are approved by the IACUC. Monkeys were fed three times daily: (a) at 09:00, each monkey received 100 g of a standard laboratory monkey maintenance diet (Lab Monkey Maintenance Diet, XTC02WC-005, with a caloric density around 4.2 kcal/g; Jiangsu Synergy Pharmaceutical Bioengineering); (b) at 14:30, each monkey was given 100 g of fresh green forage, which included root vegetables such as sweet potatoes, cassava, and pumpkin, along with various fruits and leafy vegetables, with a caloric density 0.52 kcal/g. This diet was supplemented with peanuts, sunflower seeds, unsaturated fatty acids, and vitamin E. All produce was washed and disinfected with a 0.2%−0.3% potassium permanganate solution before feeding. This feeding was conducted by the operator to enhance human-monkey interaction during other analyses; and (c) at 16:00, each monkey received another 100 g of the standard laboratory monkey maintenance diet. Water was available *ad libitum* through automatic watering devices.

To induce fatty liver, monkeys were fed an HFD for 8 months. The HFD, with a caloric density around 5.3 kcal/g, was customized by Beijing Keao Xieli Feed Co., Ltd., in accordance with the standards set forth in GB 14924.3-2010 and GB/T 18823-2010. The testing data for the batch used in this study (batch number: Y2025020704) had the following nutrient composition per kg: crude protein 174.2 g, crude fat 268 g, crude fiber 11 g, moisture 84 g, total calcium 11.3 g, total phosphorus 6.8 g, and crude ash 43 g. Each monkey received 100 g of HFD at 09:00 and another 100 g at 16:00, along with an additional 100 g of green forage at 14:30 daily. The success of fatty liver induction was assessed through histopathology (liver biopsy) and ultrasonography. The diagnostic and grading criteria were as follows:

(a) Histopathological diagnosis: A diagnosis of fatty liver was made if more than 5% of hepatocytes exhibited well-defined macrovesicular steatosis. Severity was graded as follows:

- Mild: Steatosis affecting 1/20 to 1/3 of hepatocytes

- Moderate: Steatosis involving 1/3 to 2/3 of hepatocytes

- Severe: Steatosis affecting more than 2/3 of hepatocytes

(b) Ultrasonographic criteria:

- Mild: Normal liver size at most with slightly increased echogenicity; the hepatorenal echogenicity ratio (HRR) is slightly elevated, with a uniform distribution and clear visualization of the diaphragm and intrahepatic vessels. There is no posterior attenuation.

- Moderate: Slightly enlarged liver, moderately increased echogenicity with an increased HRR, and the diaphragm and vessels are still visible but with mild posterior attenuation.

- Severe: Enlarged liver with markedly increased echogenicity (much higher than the renal cortex), poor visualization of the diaphragm and vessels, and marked posterior attenuation.

From the 21 monkeys fed an HFD, 17 were selected for the LCA intervention based on baseline liver histopathology grade, serum LDL-C level, and body weight. The 4 animals not selected had mild histopathological findings of fatty liver, with less than 5% steatosis. The demographic and baseline characteristics of the 17 enrolled animals are detailed in Supplementary Table S4.

***Anesthesia* *and* *analgesia***

For procedures requiring anesthesia (ultrasound and liver tissue biopsies), the following protocol was used. Approximately 20 min before anesthesia, monkeys were intramuscularly injected with 0.04 mg/kg of 5 mg/mL atropine to reduce respiratory secretions and prevent bradycardia. Anesthesia was performed by intramuscular injection of 7 mpk ketamine, along with etamsylate at 0.5 g per monkey, to avoid intraoperative bleeding/surgical hemorrhage. Monkeys were then transferred to the operating table, followed by connection with a veterinary anesthesia ventilator and an animal multi-parameter monitor (ZS-2000; Beijing Zhongshi Dichuang Technology) for monitoring of heart rate, respiratory rate, pulse rate, blood pressure, and pulse oximetry. Body temperature was monitored via rectal probe. Anesthesia was maintained with 1%−2% isoflurane in 100% oxygen via the ventilator.

During the post-anesthesia recovery, animals were kept warm using a heating pad and monitored continuously until sternal recumbence was regained. Body temperature and respiratory rate were recorded every 10 min during the recovery phase. After reviving, the animal was returned to its home cage and placed in right lateral recumbency to maintain an open airway, followed by clinical observation every 30 min until full recovery. Animals were then cultured regularly, while food/water intake, urination/defecation, body temperature, and general behavior were monitored daily for the following 3 days. For animals that underwent surgery, incision status was also monitored daily. The postoperative housing environment was maintained at 26°C with clean, quiet, and softly lit conditions. Any abnormal findings during the postoperative period were immediately reported to the veterinarian, who assessed the animal and prepared a treatment plan; any pharmacological intervention required prior approval from the study director and the sponsor.

For procedures that require anesthesia, such as for ultrasound and liver biopsies, the following protocol is implemented. Approximately 20 min before the administration of anesthesia, monkeys received an intramuscular injection of 0.04 mg/kg of 5 mg/mL atropine to reduce respiratory secretions and prevent bradycardia. The anesthesia itself was achieved through an intramuscular injection of 7 mpk ketamine, in conjunction with etamsylate at a dose of 0.5 g per monkey, to minimize the risk of intraoperative bleeding and surgical hemorrhage. After administration, the monkeys were transferred to the operating table and connected to a veterinary anesthesia ventilator and a multi-parameter monitor (ZS-2000; Beijing Zhongshi Dichuang Technology). This monitoring system tracked heart rate, respiratory rate, pulse rate, blood pressure, and pulse oximetry. Additionally, body temperature was monitored using a rectal probe. Anesthesia was maintained with 1%−2% isoflurane delivered in 100% oxygen through the ventilator.

During the recovery phase after anesthesia, the animals were kept warm with a heating pad and positioned in lateral recumbency. Body temperature and respiratory rate were recorded every 10 min during this period. Once the animal had revived, it was returned to its home cage and positioned in right lateral recumbency to ensure an open airway. Clinical observations were conducted every 30 min until the animal fully recovered. Following recovery, the animals were cultured regularly, and their food and water intake, urination and defecation, body temperature, and general behavior were monitored daily for 1 month. For animals that had undergone surgery, the status of their incisions was also checked daily. The postoperative housing environment was maintained at 26 °C, with clean, quiet, and softly lit conditions. Any abnormal findings during the postoperative period were immediately reported to the veterinarian, who assessed the animal and develop a treatment plan. Any required pharmacological interventions received prior approval from the study director and the sponsor.

**Formulation of LCA**

For cell-based and mouse experiments, LCA was formulated as described previously^3,30^. Briefly, for cell-based experiments, LCA powder was dissolved in DMSO to a stock concentration of 500 mmol/L, and aliquoted for storage at −20°C. The solution was placed at room temperature for 10 min (until no precipitate was visible) before adding to the culture medium. Note that any freeze-thaw cycle was not practiced to avoid the re-crystallization of LCA (otherwise forming in sheet-like, insoluble crystals) in the stock solution. For mouse experiments, LCA was coated with 2-hydroxypropyl-β-cyclodextrin before treating animals. To coat LCA, the LCA powder was dissolved in 100 mL methanol to a concentration of 0.01 g/mL, followed by mixing with 308 mL of 2-hydroxypropyl-β-cyclodextrin solution (by dissolving 2-hydroxypropyl-β-cyclodextrin in 30% (v/v, in water) methanol to 0.04 g/mL, followed by sonication for 30 min). The control vehicle was similarly prepared, with no LCA added to the 2-hydroxypropyl-β-cyclodextrin solution. After evaporating at 50℃, 90 rpm by the rotary evaporator (Rotavator R-300, Vacuum Pump V-300; BUCHI), the coated powder was stored at 4 °C for no more than 2 weeks and was freshly dissolved in free drinking water to 1 g/L before feeding mice.

For the monkey studies, LCA was formulated as a complex with phospholipid, which was then dissolved in fish oil. To prepare this complex, LCA and 1,2-distearoyl-*sn*-glycero-3-phosphocholine (DSPC) were mixed at a molar ratio of 1:1.5 (LCA:DSPC). The mixture was dissolved in tetrahydrofuran (THF) to achieve a final LCA concentration of 40 mg/mL. This solution was stirred at 400 rpm on a magnetic stirrer at 40°C. After stirring for 1 h, the solution was transferred to a rotary evaporator (Rotavapor R-300, Vacuum Pump V-300; BUCHI), where the organic solvent was removed under reduced pressure at 40°C for 50 min. The resulting concentrate was further dried in a vacuum oven (DZF-6050; Shanghai Bluepard Instruments) at 25°C for 20 h to obtain the LCA-phospholipid complex as a white or yellowish solid. This solid was then dissolved in fish oil (18/12 (EE/TG)) at a concentration of 50 mg/mL (which corresponded to the LCA content). The solution was stirred under a nitrogen atmosphere for 12 h at 25°C to yield a clear solution. After preparation, the solution was aliquoted into airtight, light-protected vials and stored at 4°C for use in monkey experiments within 2 weeks. Before oral administration to the monkeys, the formulation was allowed to equilibrate to room temperature and gently vortexed to ensure homogeneity. Doses were adjusted based on each monkey’s individual body weight, with fish oil used as a solvent to maintain consistent volume across all groups.

**Mouse GTT and ITT**

GTT and ITT were performed as described previously^3,7^. For GTT and ITT, mice were individually housed for a week prior to the experiment. For GTT, mice were fasted for 16 h (from 17:00 to 9:00 the following day), and then administered with glucose at 2 g/kg (orally gavaged or intraperitoneally injected). For ITT, mice were fasted for 6 h (8:00 to 14:00), and then 0.5 U/kg insulin was intraperitoneally injected. Blood glucose was then measured at indicated time points through tail-vein bleeding using the OneTouch UltraVue automatic Glucometer (LifeScan).

**Measurement of glucagon-like peptide-1 (GLP-1) levels**

For measuring GLP-1 levels, approximately 60 μL blood was collected (from the submandibular vein plexus) and immediately mixed with 2 μL of 50 mmol/L diprotin A (for each sample) on ice in a K_2_EDTA spray-coated tube (366420, BD P800 blood collection system). The blood samples were then centrifuged at 3,000 *g* for 10 min at 4°C, and 10 μL each was used to determine GLP-1 levels using a GLP-1 Multispecies ELISA kit according to the manufacturer’s instructions. The five-parameter logistic fitted standard curve for calculating the concentrations of GLP-1 was generated from the website of Arigo Biolaboratories (arigobio.cn/ELISA-calculator/).

**Mouse hyperinsulinemic-euglycemic clamp**

The hyperinsulinemic-euglycemic clamp was performed as described previously^3,31-33^. Briefly, mice were anesthetized with 3% isoflurane in the air via a vaporizer (R540; RWD Life Science), followed by removing the hairs and disinfecting the skin with 70% (v/v, in water) ethanol at the incision site. A small incision located approximately 5 mm superior to the sternum and 5 mm to the right of the vertical midline was made, and the fat and connective tissues beneath the pectoral muscle and surrounding the right jugular vein were gently cleaned by blunt dissection. The cephalad end of the exposed vein was then tightly tied (forming an anterior ligature) by passing a 7-0 silk suture beneath the vein. After loosely tying the caudal end of the vein via another thread of suture (posterior ligature), the vein was inserted by a 19-G needle between the two threads a few millimeters below the anterior ligature. After removing the needle, a catheter (C10PUS-MFV1610; Instech) was inserted into the vein through the needle hole, with the bevel of its tip facing towards the opening, followed by pushing forward to the caudal end for approximately 1 cm distance (until the restraining bead reaches the superior vena cava). The catheter was flushed with 100 μL heparin (200 U/mL, dissolved in saline), and then anchored by tightening the posterior ligature thread. The catheter was then tunneled (pulled with eye dressing forceps) beneath the skin from the right jugular incision to the interscapular incision (approximately 5 mm long) on the back. After exteriorizing through the interscapular incision, the catheter was connected to a Mouse Vascular Access Button (VABM1BSM-25; Instech), sealed with a protective aluminum cap (VABM1C; Instech), and secured using the 6-0 silk suture. After closing the two incisions using the 6-0 silk suture, the catheterized mouse was allowed to recover for 4 days. Mice that lost < 4% of their pre-cannulation weight after recovery were used for clamp experiments.

One day prior to the experiments, a magnetic VAB tether kit (KVABM1T/25; Instech; with its 25-G luer stub (LS25/6; Instech) replaced by a PinPor-to-Tubing Connectors (PNP3MC/25; Instech), which is connected to a PU tube (VAHBPU-T25; Instech), followed by a 4-way X connector (SCX25; Instech), and three separate luer stubs (LS25; Instech): one for infusing unlabeled glucose, one for [U-^13^C]-glucose, and the third for insulin—each connected with a PU tube) was anchored onto a counter-balanced lever arm (SMCLA; Instech), and was flushed with each perfusate by a PHD Ultra programmable syringe pump (HA3000P; Instech) in the following orders: unlabeled glucose (20% (m/v) in saline), 100 mU/mL insulin, and then 11.96 μg/μL [U-^13^C]-glucose. The mouse VAB connector on the magnetic VAB tether kit was then connected to the Mouse Vascular Access Button on the back of the catheterized mouse after the protective aluminum cap was removed. The mice were then fasted for 16 h (starting from 05:00 p.m. to 09:00 a.m. of the following day), and the experiment was performed with a two-phase protocol consisting of a 90-min equilibration period (*t* = −90 min to *t* = 0 min) and a 120-min experimental period (*t* = 0 min to *t* = 120 min). U-^13^C]-glucose was infused at 3.3 μL/min starting at *t* = *−*90 min, and was infused at a rate of 4.2 μL/min during the remaining time of the experiment. Clamping began at *t* = 0 min with a prime-continuous insulin infusion (300 mU/kg/min for 1 min), followed by a 25 mU/kg/min continuous infusion. The unlabeled glucose (20% (m/v) in saline) was then infused at 50 mg/kg/min for 10 min (from *t* = 0 min to *t* = 10 min), and the rate was adjusted according to the blood glucose level (maintained at 6−7 mmol/L; during which blood glucose was measured at every 10 min from *t* = 0 to *t* = 90 min, and every 5 min from *t* = 90 to *t* = 120 min), afterwards. At *t* = 0, 90, 100, 110, and 120 min (all at clamped state), 60 μL blood (collected from the tail vein) was taken from the tail vein. The blood was then centrifuged at 3,000 *g* for 10 min at 4°C to separate the serum. The serum ratios of [U-^13^C]-glucose to unlabeled glucose were subsequently determined using HPLC-MS.

For HPLC-MS, 10 μL serum was mixed with 1 mL pre-cooled methanol:acetonitrile 1:1 (v/v) solution, followed with 20 s of vortexing. After centrifugation at 15,000 *g* for another 15 min at 4°C, 800 μL aqueous phase was collected. The sample was then lyophilized in a vacuum concentrator (CentriVap Benchtop Centrifugal Vacuum Concentrator (#7310037; Labconco), equipped with a CentriVap −84°C Cold Trap (#7460037; Labconco) and an EDWARDS nXDS15i pump) at 4°C for 12 h, and then dissolved in 50 μL of 50% (v/v, in water) acetonitrile, followed by centrifugation at 15,000 *g* for another 30 min at 4°C. A total of 20 µL supernatant was loaded into an injection vial (4025BS-629-100; ANPEL Laboratory Technologies) equipped with a snap cap (5397-09FRB-100; ANPEL Laboratory Technologies), and 2 μL supernatant was injected into a HILIC column (ZICpHILIC, 5 μm, 2.1 mm × 100 mm, PN: 1.50462.0001; Millipore) on an ExionLC AD UPLC system (SCIEX), which is interfaced with a QTRAP 5500 MS (SCIEX). The mobile phase consisted of 15 mmol/L ammonium acetate containing 3 mL/L ammonium hydroxide (> 28%, v/v) in the LC-MS grade water (mobile phase A), and LC-MS grade 90% (v/v) acetonitrile in LC-MS grade water (mobile phase B), and was run at a flow rate of 0.2 mL/min. The HPLC gradient elution program was: 95% B held for 2 min, then to 45% B in 13 min, held for 3 min, and then back to 95% B for 4 min. Each sample was analyzed in the negative mode on the HPLC-MS. The MS was run on a Turbo V ion source with spray voltages of −4,500 V, source temperature at 550°C, Gas No.1 at 50 psi, Gas No.2 at 55 psi, and curtain gas at 40 psi. Glucose levels were measured using the multiple reaction monitoring (MRM) mode, and both the declustering potential and collision energy were optimized using a glucose standard prior to each experiment. Data were collected using Analyst software (v.1.7.1, SCIEX), and the relative amounts of metabolites were analyzed using MultiQuant software (v.3.0.3, SCIEX). Levels of [U-^13^C]-glucose and unlabeled glucose were measured using full scan mode with m/z values of 185.0/92.0 for [U-^13^C]-glucose, and 179.1/89.1 for unlabeled glucose. The resting hepatic glucose output rate (HGP) was calculated by dividing the resting-state infusion rate of [U-^13^C]-glucose with the ratio of [U-^13^C]-glucose to unlabeled glucose, while the clamped HGP was calculated by dividing the value of differences between the average, clamp-state infusion rate of the unlabeled glucose and the [U-^13^C]-glucose with the ratio of [U-^13^C]-glucose to unlabeled glucose. The glucose disposal rate during clamping was the sum of the infusion rates of [U-^13^C]-glucose or unlabeled glucose, plus the clamped HGP value.

**Histology**

For hematoxylin and eosin (H&E), Sirius Red, and TUNEL staining, liver tissues were quickly excised from mice after sacrifice, while liver tissues from monkeys were obtained via liver puncture (as detailed in the “Assessment of physiological indexes in monkeys” section). Tissues were then fixed in 4% (v/v) paraformaldehyde for 24 h at room temperature and transferred to embedding cassettes. The cassettes were then rinsed with running water for 2 h at room temperature, followed by successive soaking for 1 h each in 70% ethanol (v/v in water), 80% ethanol, and 95% ethanol. The fixed tissues were further dehydrated in anhydrous ethanol for 30 min twice, then immersed in 50% xylene (v/v in ethanol) for 30 min, followed by two changes of xylene, 15 min each, and two changes of paraffin wax (58−60°C), each 1.5 h. The dehydrated tissues were embedded in paraffin on a HistoCore Arcadia Paraffin Embedding Machine (Leica), sectioned into 5-μm slices with a HistoCore BIOCUT manual rotary microtome (Leica), and then mounted on glass slides.

H&E staining was performed as described previously^34^, with minor modifications. Briefly, the sections were baked at 45°C overnight, and subsequently baked at 65°C for 30 min. After deparaffinization in two changes of xylene, 5 min each, sections were rehydrated through a descending ethanol series: two changes of anhydrous ethanol, 1 min each, 95%, 85%, and 75% ethanol, 3 min each, followed by washing in running water for 1 min. The rehydrated sections were stained in Mayer’s hematoxylin solution for 5 min, differentiated in 1% (v/v, hydrochloric acid in ethanol) acid alcohol for 30 s, washed in running water for 10 min, and then stained in eosin Y-solution for 1 min. The stained sections were dehydrated twice in 95% ethanol, 1 min each, twice in anhydrous ethanol, 1 min each, and twice in xylene, 4 min each. The stained sections were mounted with Canada balsam and visualized on an AxioScan 7 scanner (Zeiss). Images were processed and analyzed using Zen 3.4 software (Zeiss) and were formatted using Photoshop 2023 software (Adobe).

The nonalcoholic fatty liver disease [NAFLD] activity score (NAS) and the histological scores for steatosis grade, hepatocellular ballooning, and lobular inflammation were determined from microscopic images of H&E-stained liver sections from HFD mice, as described previously^14,35^. Briefly, steatosis grade was determined by the percentage of lipid droplets within the total tissue area, lobular inflammation by the number of inflammatory foci, and hepatocellular ballooning by the number of ballooned hepatocytes. NASs were obtained from the sum of the above three scores.

Sirius Red staining was performed as described previously^6^, with minor modifications. Briefly, the sections were baked overnight at 45°C, and subsequently at 65°C for 2 h. After deparaffinization in three changes of xylene, 5 min each, sections were rehydrated through a descending ethanol series: two changes of anhydrous ethanol, 3 min each, 95%, 85%, and 75% ethanol, 5 min each, followed by washing in running water for 5 min. The sections were stained in Sirius Red solution for 2 h, washed in running water for 1 min, and then stained in eosin Y solution for 10 min. After differentiated in 1% (v/v, hydrochloric acid in ethanol) acid alcohol for 30 s, washed in running water for 10 min, and then dehydrated through 75%, 85%, 95% and anhydrous ethanol, 2 min each, the stained sections were cleared twice in xylene, 5 min each. The stained sections were mounted with Canada balsam and visualized on an AxioScan 7 scanner (Zeiss). Images were processed and analyzed using Zen 3.4 software (Zeiss) and were formatted by Photoshop 2023 software (Adobe).

TUNEL staining was performed using the TUNEL Apoptosis Detection Kit as described previously^7^, with minor modifications. Briefly, the sections were baked overnight at 45°C, followed by 2 h at 65°C. After deparaffinization in three changes of xylene for 5 min each, the sections were rehydrated through a descending ethanol series: anhydrous, 95%, 85%, and 75% ethanol, 3 min each, followed by three washes with PBS for 5 min each. The sections were then washed twice with PBS for 5 min each and permeabilized with a proteinase K solution (20 μg/mL in PBS, freshly diluted from a 2 mg/mL stock) for 20 min at room temperature. After three PBS washes for 5 min each, excess liquid was carefully removed from around the samples using filter paper, and a hydrophobic barrier was drawn around each section using a PAP pen. The sections were maintained in a humidified chamber to keep them moist for the following steps. Each section was incubated with 50 μL freshly prepared 1× Equilibration Buffer (diluted from a 5× Equilibration Buffer in double-distilled water) at room temperature for 30 min. Following this, the sections were incubated in a dark, humidified chamber at 37°C for 1 h with 50 μL TdT Reaction Buffer (freshly prepared by mixing double-distilled water, 5× Equilibration Buffer, FITC-12-dUTP Labeling Mix, and Recombinant TdT Enzyme in a ratio of 34:10:5:1). After the incubation, the sections were washed three times with PBS for 5 min each and then incubated for 5 min at room temperature in the dark with a freshly prepared DAPI solution (2 μg/mL in PBS). The slides were rinsed three times with double-distilled water for 5 min each at room temperature. The drained sections were mounted with an anti-fade mounting medium containing DAPI (DAPI Fluoromount-G^TM^) and imaged using an Axioscan 7 digital slide scanner (Zeiss).

For immunohistochemistry staining of F4/80, Ly6G, and cleaved caspase-3, liver tissues were fixed, dehydrated, embedded, sectioned, and rehydrated following the same steps as for H&E staining. The sections were incubated in an antigen retrieval solution (10 mmol/L sodium citrate, pH 9, in PBS) and heated to boiling for 20 min. After cooling to room temperature, the sections were washed three times with PBST (0.1% (v/v) Tween-20 in PBS), 3 min each, followed by blocking with BSA solution (PBS containing 5% (m/v) BSA) for 1 h at room temperature. The sections were then incubated with the antibody solution (50 µL per section; 1:500 dilution for F4/80 antibody, 1:300 for Ly6G, and 1:400 for cleaved caspase-3, all diluted in BSA solution) for 12 h at 4°C. The sections were next washed three times with PBST for 5 min each at room temperature, followed by incubation with Alexa Fluor 647-conjugated goat anti-rabbit IgG secondary antibody (50 µL per section; 1:200 in BSA solution) for 2 h at 4°C in a dark, humidified chamber. After this, the sections were washed three times with PBST for 5 min each at room temperature, then incubated with Hoechst (50 µL per section, diluted to 0.4 µg/mL in BSA solution) for 15 min. The sections were washed three times with PBST for 5 min each at room temperature and mounted with 90% glycerol. Images were taken, processed, and analyzed using Zen 3.4 software (Zeiss), and formatted with Photoshop 2023 software (Adobe).

**Mouse serum AST and ALT**

Serum AST and ALT levels were measured as described previously^6,7^, with minor modifications. Briefly, approximately 100 μL blood was collected and allowed to sit at room temperature for 20 min. The blood was then centrifuged at 3,000 *g* for 10 min at 4°C. A total of 5 μL serum was used to measure ALT and AST levels using the Alanine Aminotransferase Assay Kit and the Aspartate Aminotransferase Assay Kit, respectively, in accordance with the manufacturer’s instructions. The optical density (OD; recorded with a SpectraMax M5 microplate reader (Molecular Devices) using SoftMax Pro software (v5.4.1.1, Molecular Devices)) of each sample was calculated by subtracting the value obtained from the testing well—where the serum sample was mixed with the reaction buffer and then the termination buffer—from the value obtained from the blank well—where the serum sample was added after mixing the reaction and termination buffers. These OD values were then plotted against Karmen units to create a standard curve, from which the activities of the sample ALT and AST were determined.

**Mouse primary hepatocytes**

Mouse primary hepatocytes were isolated with a modified two-step perfusion method using Liver Perfusion Media and Liver Digest Buffer as described previously^16^. For isolation of hepatocytes, mice were first anesthetized; and an I.V. catheter (0.72 mm × 19 mm) was inserted into the postcava. After cutting off the portal vein, mice were perfused with 50 mL Liver Perfusion Media at a rate of 5 mL/min, followed by 50 mL Liver Digest Buffer at a rate of 2.5 mL/min. The digested liver was then briefly rinsed with PBS, and then dismembered by gently tearing apart the Glisson’s capsule with two sterilized, needle-pointed tweezers on a 6-cm dish containing 3 mL PBS. The dispersed cells were mixed with 10 mL ice-cold William’s medium E plus 10% FBS, and were filtered by passing through a 100-μm Cell Strainer (352360; Falcon). Cells were then centrifuged at 50 *g* at 4°C for 2 min, followed by washing twice with 10 mL ice-cold William’s medium E plus 10% FBS. Cells were then immediately plated (at 60%−70% confluence) in collagen-coated 6-well plates in William’s medium E supplemented with 10% FBS, 100 IU penicillin, and 100 mg/mL streptomycin, and were maintained at 37°C in a humidified incubator containing 5% CO_2_. After 4 h of attachment, the medium was replaced with fresh William’s medium E with 1% (w/v) BSA for another 12 h before further use.

**Measurement of TAG levels in mouse liver tissues and cultured hepatocytes**

TAG levels were measured as described previously^36,37^, with minor modifications. All procedures were performed on ice unless otherwise noted. For cultured hepatocytes, the cells were washed three times with PBS, and then scraped into ice-cold tubes and centrifuged at 500 *g* for 5 min at 4°C. The pellet was resuspended in 500 μL isopropanol, vortexed, and centrifuged at 12,000 *g* for 5 min at 4°C. The supernatant was used for TAG measurement, while the pellet was air-dried and subsequently dissolved in 1 mL of 0.2 mol/L KOH overnight on a shaker for protein quantification via a Coomassie Brilliant Blue G-250 assay using BSA standards. For liver tissues, approximately 50 mg tissues (exact wet weight recorded) were homogenized in 500 μL isopropanol with steel beads in an ice bath. The homogenate was then centrifuged at 12,000 *g* for 5 min at 4°C, and the supernatant was collected.

TAG levels in the cell or liver supernatants were determined using the Amplex Red Triglyceride Assay Kit. The assay buffer was equilibrated to room temperature, and the TAG standard (50 mmol/L) was incubated at 80°C for 15 min, with brief mixing to ensure complete dissolution. A working solution was freshly prepared by mixing 44 μL assay buffer, 2 μL Amplex Red, 2 μL Enzyme Mix (briefly centrifuged and mixed), and 2 μL Cofactor per 50 μL reaction, and keeping on ice in the dark. For sample measurement, 10 μL hepatocyte supernatant or 2.5 μL liver supernatant was added to each well of a clear 96-well plate. The volume in each well was brought to 50 μL with assay buffer. A standard curve was created by diluting the TAG standard (1:4 dilution of the 50 mmol/L stock with assay buffer) to final concentrations of 0, 0.1, 0.2, 0.5, 1, 2, 5, and 10 mmol/L in 50 μL assay buffer. Lipase solution (2 μL) was then added to each well and mixed, and the plate was incubated at 37°C for 20 min. Blank wells contained only the assay buffer, while sample background control wells received 2 μL assay buffer instead of the Lipase solution. Afterward, 50 μL working solution was added to each well and mixed, and the plate was incubated at 37°C for 60 min in the dark. The OD was measured at 570 nm. TAG concentrations were calculated from the standard curve and multiplied by the dilution factor. For liver samples, TAG content was normalized to tissue wet weight; for hepatocytes, TAG content was normalized to total protein (in mg) determined by the G-250 assay.

**Assessment of physiological indexes in monkeys**

***Body weight, abdominal circumference, subcutaneous fat thickness, and BMI***

Body weight was recorded using an electronic scale (YP20000; Shanghai Yueping Scientific Instrument; capacity 20 kg, precision 1 g). The measurement procedure was as follows. First, a transfer cage and an animal neck collar were placed on a foam pad on the scale, and the scale was tared once the reading stabilized. Next, the collar was removed, and the animal’s identification was verified. The transfer cage was then secured to the animal’s home cage door, and the doors were opened to gently guide the animal into the transfer cage. After closing the transfer cage door, the animal was weighed to the nearest 0.01 kg, and the weight was recorded. Finally, the animal was returned to its home cage.

Abdominal circumference and subcutaneous fat thickness were measured under anesthesia. An assistant lifted the anesthetized animal’s arm and positioned it in a seated posture on the operating table. The abdominal circumference was recorded by wrapping a flexible measuring tape around the abdomen at the level of the umbilicus. To measure subcutaneous fat thickness, a skinfold caliper (PZJ-01; Changzhou Zefeng) was applied horizontally to the skinfold pinched about 1 cm below the umbilicus using the thumb and index finger. The measurements were repeated three consecutive times, allowing for a tolerance of less than 5% between readings, and the mean value was calculated.

BMI was calculated by dividing body weight (in kg) by the square of the crown-rump length (in cm^2^) and then multiplying the result by 10,000. The crown-rump length was measured at baseline under anesthesia, extending from the top of the head to the base of the tail. Since the animals were adults, this length was assumed to remain constant throughout the study, so the baseline measurement was used for all BMI calculations.

***Blood pressure***

Blood pressure was measured using a non-invasive veterinary electronic sphygmomanometer (CONTEC08A-VET; Contec Medical Systems). All measurements were performed on awake, restrained animals, with the right upper arm serving as the measurement site. The procedure was as follows:

(a) Preparation. Before measurements, the battery and time settings of the sphygmomanometer were checked, and a medium-sized cuff was connected to the device. The animal’s fur over the measurement site was shaved in an area larger than the cuff.

(b) Animal positioning and cuff placement. A soft towel was placed on the armrest of a monkey chair before positioning the animal. After moving and securing the animals in the monkey chair, the shaved upper arm was gently held in extension and padded with a soft cushion for comfort. The animal was soothed and allowed to relax before wrapping the cuff around the arm, aligning it at the same level as the heart, with the lower edge of the cuff positioned 1−2 cm above the antecubital fossa. The cuff tubing was adjusted to sit over the brachial artery, and the tightness was modified to allow one finger to fit comfortably between the cuff and the arm.

(c) Measurement. Measurements were taken in accordance with the manufacturer’s standard operating procedure. The “start” button was pressed to initiate the measurement, and systolic and diastolic pressures (in mmHg) were displayed upon completion. Three consecutive measurements were recorded at each time point, and the average of the three measurements was used for analysis. If any measurement deviated from the median by more than 10 mmHg, additional readings were taken. If the blood pressure value differed from the previous measurement by more than 10 mmHg, the entire measurement process was repeated.

(d) Post-measurement procedures. After the measurement, the animal was returned to its home cage and given a small treat (such as peanuts or sunflower seeds) as positive reinforcement.

***Food*** ***intake***

Daily food intake was calculated based on the following observations.

(a) Morning observation (08:00). During the inspection of daily animal health and well-being, the leftover HFD from the previous evening (found both in the food hopper and on the cage floor) was weighed and recorded. After the cage cleaning (took place around 8:30), the remaining food was discarded. During this cleaning, the floor of each monkey’s cage was flushed, and the feed box was wiped dry with a towel.

(b) Morning feeding (09:00). Each animal received approximately 100 g of HFD, which was weighed using an electronic scale (YP20000; Shanghai Yueping Scientific Instrument). The exact amount of food was recorded.

(c) Green forage feeding (14:30). Fresh green forage, consisting of fruits and vegetables (approximately 100 g per animal), was provided. The exact amount of food was recorded.

(d) Evening feeding (16:00). The leftover green forage from the afternoon was measured (during the afternoon animal health check), after which each animal was given approximately 100 g of HFD. The precise amounts were recorded. For animals that occasionally consumed less HFD, supplemental foods, such as steamed buns (with a calorie density of approximately 2.2 kcal/g) or a standard maintenance diet, were offered to ensure their health. It should be noted that these supplements were not included in the analysis of food consumption since they have a much lower caloric density than HFD, and their occurrence in our animal cohorts was rare (see Supplementary Table S3 for details).

The daily HFD intake was calculated by subtracting the post-feeding weight from the pre-feeding weight, including any food spilled onto the cage floor. In this study, it was observed that the monkeys completely consumed the green forage each day, and their daily green forage intake was recorded as 100 g. Average daily food intake over the week was calculated for each animal, and total calorie intake was computed based on the average calorie densities of each type of diet.

***Body fat composition***

Total body fat percentage was assessed using dual-energy X-ray absorptiometry (DEXA; Lunar Prodigy; GE Healthcare). Prior to the scan, the animals were fasted for 8 h (from 10:30 to 18:30). They were then anesthetized following the protocol described in the “Anesthesia and analgesia” section, but with the use of Zoletil 50 (3.5 mg/kg, intramuscular, single dose) instead of ketamine, as Zoletil 50 provides better muscle relaxation and sedation. In addition, isoflurane maintenance was not required for this procedure.

During the DEXA scan, the animals lied in a supine recumbent position, with their limbs extended and secured using non-metallic tape. The scanner settings used were: mode “Child - Whole Body - Small”; scan length of 100.9 cm; and scan width of 40.1 cm. The sex parameter was set to “Male.” A quality control phantom was used daily to calibrate the DEXA instrument before any scans took place. Data analysis was conducted using enCORE software, and all animal positioning and data analysis were performed by the same investigator. The actual scan was acquired by a licensed radiology technician at the collaborating hospital. Although different technicians were involved at various time points, all held the necessary professional certification. The outcome reported in this study was the total body fat percentage, calculated as (total fat mass/total mass) × 100.

***Ultrasound assessment of the liver***

Hepatic ultrasonography was conducted using a laboratory-dedicated Doppler ultrasound system (CR9; Pretta Ultrasound) equipped with a high-frequency linear probe (5−12 MHz). Before scanning, the animals were fasted for 12 h and anesthetized according to the protocol described in the “Anesthesia and analgesia” section. The animals were positioned in dorsal recumbency; when necessary, they were also placed in left lateral recumbency to optimize the acoustic windows. The right intercostal, subcostal, and subxiphoid approaches were utilized to obtain multiple scan planes of the liver. The following sonographic parameters were measured for each animal.

(a) Liver size. Liver size was assessed qualitatively for hepatomegaly by measuring the craniocaudal length of the right lobe in the mid-clavicular line. An adult male cynomolgus monkey (approximately 10 kg body weight) with a liver thickness greater than 6 cm (normal range: approximately 4−5 cm) was suspected of having hepatomegaly.

(b) Hepatic echogenicity. This was compared with the echogenicity of the renal cortex in the same sonographic window. A diagnosis of fatty liver was made when liver echogenicity was equal to or greater than that of the renal cortex, accompanied by posterior attenuation (reduced depth of echoes) and blurring of the hepatic vasculature and diaphragm margins.

(c) Distribution of fatty infiltration. This was classified as diffuse, focal, segmental, or inhomogeneous based on the pattern of increased echogenicity.

(d) Color Doppler flow imaging (CDFI). CDFI was performed to differentiate blood vessels from bile ducts (not used as a criterion for assessing the severity of fatty liver).

Fatty liver severity was graded semi-quantitatively by two independent radiologists who performed blinded evaluations, unaware of the treatment group and time point. The grading criteria were described in the “Monkey enrollment and fatty liver induction” section. All ultrasound examinations were conducted by the same experienced sonographer. Images were independently stored and reviewed by the two certified radiologists, who were not the same as the sonographer; any disagreements were resolved by consensus.

***Blood sample collection and processing***

Before each blood collection, animals were fasted overnight for approximately 15 h (from 17:00 to 08:00 the next day). All blood collections were performed between 08:00 and 10:00. For each blood collection time point, blood was drawn from a superficial vein in either the upper or lower limb using a sterile needle and Vacutainer collection tubes (BD). Two types of collection tubes were used.

(a) Vacutainer SST Tubes (367986; BD). These tubes were used for serum biochemistry. Approximately 2 mL whole blood was collected from each animal. The tubes were kept at room temperature for 60 min to allow for clotting, and then centrifuged at 4°C for 10 min at 4,000 rpm (using a CLT55R centrifuge, Hunan Xiangyi Laboratory Instrument) in No. 3 rotor. Serum was aliquoted into two cryovials (0.5 mL each) and stored at −80°C until analysis.

(b) K2EDTA Blood Collection Tubes (367856; BD). These tubes were used for hematology (complete blood count) and for isolating plasma to measure leptin levels. Again, approximately 2 mL whole blood was collected from each animal. The tubes were gently inverted to mix, providing whole blood sample for further analysis. For plasma collection, the mixture was centrifuged at 4°C for 10 min at 4,000 rpm. The plasma was aliquoted into cryovials (0.5 mL per vial) and stored at −80°C until analysis.

For serum biochemistry and hematology, freshly collected whole blood was placed in a transport box with ice packs and sent to the external testing facility, Guangxi International Zhuang Medicine Hospital. All samples were collected within 1 h (starting around 10:00 and completed within 60 min). The transport time from the facility to the hospital was less than 30 min. Upon arrival, the hospital initiated sample analysis within 1 h. For samples sent to Xiamen University for the analysis of LCA concentrations, serum was shipped on dry ice and remained frozen throughout transit.

***Serum biochemistry and hematology***

Serum biochemical parameters were measured using an automated analyzer (Architect C16000; Abbott) at Guangxi International Zhuang Medicine Hospital, with technicians blinded to the treatment group. The following parameters were assessed.

(a) Liver function: albumin (ALB), total bilirubin (TBIL), alanine aminotransferase (ALT, weekly), aspartate aminotransferase (AST, weekly), cholinesterase (CHE), and total bile acids (TBA).

(b) Kidney function: blood urea (UREA) and creatinine (Cr).

(c) Lipid profile: total cholesterol (TC, weekly), triglycerides (TAG, weekly), high‑density lipoprotein cholesterol (HDL‑C, weekly), and low‑density lipoprotein cholesterol (LDL‑C, weekly).

(d) Glucose metabolism parameters: fasting blood glucose (FBG, weekly) and glycated hemoglobin (HbA1c).

(e) Other: testosterone (T).

Hematology was performed using an automated hematology analyzer (XN-10; Sysmex) at the same hospital, again with technicians blinded to the treatment group. The following parameters were measured: white blood cell count (WBC) and differential (neutrophils, lymphocytes, monocytes, eosinophils, basophils – both absolute counts and percentages); red blood cell count (RBC); hemoglobin (HGB); hematocrit (HCT); mean corpuscular volume (MCV); mean corpuscular hemoglobin (MCH); mean corpuscular hemoglobin concentration (MCHC); red cell distribution width (RDW‑SD and RDW‑CV); platelet count (PLT); plateletcrit (PCT); mean platelet volume (MPV); platelet distribution width (PDW); and platelet-large cell ratio (P‑LCR).

All assays were performed on fresh whole blood using the same analyzer for all time points, with each sample run in singlicate.

***Plasma leptin ELISA***

Plasma concentrations of leptin were measured using Monkey Leptin ELISA Kit. The procedure is as follows.

(a) All reagents and samples were brought to room temperature and equilibrated for 60 min.

(b) Standard wells (for serial dilutions of the standard), blank wells, and sample wells were prepared on the provided 96-well plates. A total of 50 μL standard or sample were added to the respective wells, while the blank wells received no liquid.

(c) A total of 100 μL HRP-conjugated detection antibody was added to each well, except for the blanks. The plates were sealed and incubated at 37°C for 60 min.

(d) The wells were washed five times with 350 μL wash buffer per well, decanting the solution each time and tapping the plate dry on absorbent paper.

(e) A total of 50 μL each of substrate A and substrate B were added to each well, and the plates were incubated at 37°C in the dark for 15 min.

(f) A total of 50 μL stop solution was added to each well, and the OD value was measured at 450 nm using a microplate reader (Infinite F50; TECAN) within 15 min.

A standard curve was generated by plotting OD values against known standard concentrations using a four-parameter logistic curve fit. Sample concentrations were interpolated from the standard curve after subtracting the blank OD. All samples were run in duplicate, and the mean value was used for analysis.

***Liver biopsy***

Liver biopsies were performed under general anesthesia and in aseptic conditions. The animals were fasted for 12 h prior to the surgery. Anesthesia was performed as described in the “Anesthesia and analgesia” section. To reduce the risk of bleeding before the incision, etamsylate (0.5 g per animal) was given intramuscularly. The ultrasound-guided percutaneous liver biopsy was conducted using a disposable 16G × 16 cm biopsy needle (MN1616; Bard Magnum) mounted on a spring-loaded biopsy gun (MG1522; Bard Magnum). The specimen notch length was set to 2.2 cm, consistent for all animals and both time points. The procedure included the following steps.

(a) A high-frequency ultrasound probe was sterilized with 75% alcohol and covered with a sterile sheath. The liver was scanned to identify a safe puncture site, avoiding large blood vessels, the gallbladder, and the bile duct. The target site was consistently chosen in the right posterior lobe of the liver, approximately at the intersection of the last rib margin and the nipple line. However, the exact puncture point could be adjusted slightly (anterior or posterior) based on real-time B-mode imaging to avoid visible blood flow.

(b) The biopsy needle was advanced through the skin at the marked site under real-time ultrasound guidance. The needle tip was slowly advanced until it came into contact with the liver capsule.

(c) Once the needle tip was in the target area of the liver (while avoiding the diaphragm and major vessels), the trigger of the biopsy gun was activated to obtain a core of liver tissue.

(d) After withdrawing the needle, sterile gauze was pressed over the puncture site for 5 min. An ultrasound was immediately repeated to confirm the absence of anechoic fluid to determine whether active bleeding occurred.

(e) The liver tissue core was immediately placed into 4% paraformaldehyde for fixation, as detailed in the “Histology” section for further analysis.

Following the completion of the biopsy procedure, anesthesia was discontinued. The animal was placed in a warm recovery cage and monitored until it regained sternal recumbency and normal responsiveness. Postoperative clinical observations were performed according to the protocol outlined in the “Anesthesia and analgesia” section. During the recovery phase, analgesia and antibiotic therapy were provided for 3 days. Tramadol hydrochloride (15 mg per animal, intramuscular) and ampicillin sodium (250 mg per animal, intramuscular) were administered twice daily. The incision site was examined daily for signs of infection, such as redness, swelling, or discharge, and was disinfected with a 0.5% iodophor solution once daily for 3 days post-surgery. Any animal showing signs of pain (e.g., hunched posture, reduced appetite, or vocalization) received additional analgesia. In this study, no instances of wound dehiscence or infection were observed in any animal.

**Cell lines**

In this study, none of the cell lines used are listed among the known misidentified cell lines maintained by the International Cell Line Authentication Committee (https://iclac.org/databases/cross-contaminations/). HepG2 (#HB-8065) cells were purchased from ATCC, while HepaRG (#IM-H415) cells were purchased from IMMOCELL. All cells were verified to be free of mycoplasma contamination and authenticated through STR sequencing. HepG2 cells were maintained in DMEM supplemented with 10% FBS, 100 IU penicillin, and 0.15 mg/mL streptomycin. HepaRG cells were kept in HepaRG cell medium (#IM-H415-1; IMMOCELL). All cells were cultured at 37°C in a humidified incubator with 5% CO_2_.

**Reagents**

The following antibodies were purchased from Cell Signaling Technology: rabbit anti-phospho-AMPKα-Thr172 (#2535, RRID: AB_331250; 1:1,000 for IB), anti-AMPKα (#2532, RRID: AB_330331; 1:1,000 for IB), anti-phospho-ACC-Ser79 (#3661, RRID: AB_330337; 1:1,000 for IB), anti-ACC (#3662, RRID: AB_2219400; 1:1,000 for IB), anti-phospho-RPA32/RPA2-S8 (#54762, RRID: AB_2799471; 1:1,000 for IB); anti-RPA32/RPA2 (#35869, RRID: AB_2799086; 1:1,000 for IB); anti-histone H3 (#4499, RRID: AB_10544537; 1:1,000 for IB); anti-cleaved caspase-3 (#9664, RRID: AB_2070042; 1: 400 for IF); anti-Ly-6G (#87048, RRID: AB_2909808; 1:300 for IF). The following antibodies were purchased from Abcam: rabbit anti-gamma H2A.X (#ab11174, RRID: AB_297813; 1:1000 for IB), anti-F4/80 (#ab111101, RRID: AB_10859466; 1:500 for IF). The goat anti-rabbit IgG (H+L) Highly Cross-Adsorbed Secondary Antibody, Alexa Fluor™ Plus 647 (#A32733, RRID: AB_2633282; 1:200 for IF) was purchased from Thermo. The horseradish peroxidase (HRP)-conjugated goat anti-rabbit IgG (#111-035-003, RRID: AB_2313567; 1:5,000 dilution for IB) antibody was purchased from Jackson ImmunoResearch.

The following reagents were purchased from Sigma (catalog numbers in parentheses, the same as below): DMSO (D2650), LCA (L6250), 2-hydroxypropyl-β-cyclodextrin (C0926), NaCl (S7653), streptomycin (85886), glucose (G7021), haematoxylin solution (51275), heparin (H3149), paraformaldehyde (158127), eosin Y-solution (318906), methanol (646377), ethanol (459836), chloroform (C7559), PBS (P5493), Triton X-100 (T9284), xylene (534056), d-mannitol (M4125), acetonitrile (34888), Canada balsam (C1795), BSA (A2153), glycerol (G5516), NaOH (S8045), isopropanol (34863), EDTA (E6758), EGTA (E3889), MgCl_2_ (M8266), KCl (P9333), IGEPAL CA-630 (NP-40; 3021), dithiothreitol (DTT; 43815), agarose (A9539), cholic acid-2,2,3,4,4-d5 (CA-d5; 614106), ammonium formate (70221), lithocholic acid-2,2,4,4-d4 (LCA-d4; 589349), Trizma base (Tris; T1503), sodium pyrophosphate (P8135), β-glycerophosphate (50020), SDS (436143), glutaraldehyde solution (G5882), glycine (G8898), K_3_Fe(CN)_6_ (455946), acetone (534064), sodium citrate (71497), sodium azide (NaN3; S2002), gentamycin (345814), collagenase A (11088793001), Tween-20 (P9416), NaH_2_PO_4_ (S8282), formaldehyde solution (formalin; F8775), ampicillin (A9518), Nile Red (72485), formic acid (5.43804), oleic acid (OA, O1383), Hoechst (B2261), tetrahydrofuran (THF, 401757), ammonium hydroxide (338818), Paraformaldehyde (158127), hydrochloric acid (258148), proteinase K (V900887), isopropanol (34863), KOH (221473), SDS (436143), ammonium formate (70221), and formic acid (5.43804). The following reagents were purchased from Thermo: DMEM-high glucose (12800082), Liver Perfusion Medium (17701038), Liver Digest Medium (17703034), William’s E Medium (12551032), DMEM without phenol (31053028), MEM non-essential amino acids solution (11140050), GlutaMAX (35050061), sodium pyruvate (11360070), Trypan Blue Stain (T10282), FBS (10099141C), penicillin–streptomycin (15140163), BODIPY (D3922), Prestained Protein MW Marker (26612), BCA Protein Assay kit (A55865), and GLP-1 Multispecies ELISA Kit (BMS2194). Protease inhibitor cocktail (70221) was purchased from Roche. [U-^13^C]-glucose (CLM-1396-PK) and [U-^13^C]-glutamine (CLM-1822-H-PK) were purchased from Cambridge Isotope Laboratories. DAPI solution (40728ES03) and DAPI Fluoromount-G (36308ES20) were purchased from Yeasen. Coomassie Brilliant Blue G-250 (A600038) was purchased from Sangon Biotech. Nonfat dry milk (9999) was purchased from Cell Signaling Technology. Insulin (Novolin R) was purchased from Novo Nordisk. Tamoxifen (HY-13757A) was purchased from MCE. WesternBright ECL and peroxide solutions (210414-73) were purchased from Advansta. 1,2‑distearoyl‑sn‑glycero‑3‑phosphocholine (DSPC; S01005) was purchased from AVT (Shanghai) Pharmaceutical Tech Co., Ltd. Fish oil (18/12 (EE/TG); containing 160 mg/g EPA and 100 mg/g DHA) was customized from Shandong Yuwang Pharmaceutical. Amplex Red Triglyceride Kit (S0219S) was purchased from Beyotime. Alanine aminotransferase Assay Kit (C009-2-1) and Aspartate aminotransferase Assay Kit (C010-2-1) were purchased from Nanjing Jiancheng Biotechnology Research Institute. TUNEL Apoptosis Detection Kit (40307ES60) and Annexin V-FITC/PI Apoptosis Detection Kit (40302ES60) were purchased from Yeasen. Monkey Leptin ELISA Kit (ml023356V) was purchased from Shanghai Enzyme-linked Biotechnology Co., Ltd. Sirius Red solution (SS02120) was purchased from Fuzhou Xichen Technology.

The following reagents were specifically used in monkey experiments, with the corresponding approval numbers from the Chinese Ministry of Agriculture and Rural Affairs or the National Medical Products Administration of China in parentheses: ketamine hydrochloride (100761663) was purchased from Jiangsu Zhongmu Beikang Pharmaceutical, atropine sulfate (120192742) was purchased from Hefei Dubang Bio-pharmaceutical, isoflurane (101357015) was purchased from H.F.Q. Bio-technology, and etamsylate (H37022829) was purchased from Shandong Fangming Pharmaceutical. The iodophor solution (disinfection product number of Shandong province: (2002)0059) was purchased from Shandong Lierkang Disinfection Technology.

**Confocal microscopy**

Lipid droplets in hepatocytes were imaged by confocal microscopy as described previously^38-40^, with minor modifications. In brief, cells were seeded in 35-mm glass-bottom dishes (D35-20-10-N; In Vitro Scientific) and grown to 50%−70% confluence. Cells were then incubated with 250 μmol/L OA for 12 h. Following this, the cells were washed twice with PBS and incubated in fresh medium containing 1 μmol/L LCA for an additional 4 h. After washing twice with PBS, the cells were treated with 10 μmol/L BODIPY and 1 μg/mL Hoechst for 20 min. The cells were then washed twice with PBS and placed in fresh medium containing 1 μmol/L LCA. During imaging, cells were maintained at 37°C and 5% CO_2_ in a humidified incubation chamber (Incubator PM S1; Zeiss). Images were captured using a Zeiss LSM 980 microscope, with BODIPY and Hoechst excited by a 488-nm diode laser (ex-fiber power: 15 mW) and a 405-nm diode laser (ex-fiber power: 10 mW), respectively, using a 63×, 1.4 NA oil objective.

Lipid droplet quantification was performed using ImageJ (Fiji) software. Prior to quantification, lipid droplets in each cell from the captured images were segmented using “regions of interest (ROIs)” that were automatically generated by the deep-learning-based algorithm Cellpose^41^ (set up via Anaconda Prompt, according to the instructions; https://github.com/MouseLand/cellpose). To delineate the ROI for each lipid droplet, the images from the BODIPY channel were exported as 16-bit .tif files. A medium diameter for lipid droplets, estimated to be 20 pixels in this study based on the typical size observed in the images, was assigned to the algorithm. The algorithm was then executed, and the resulting ROIs were illustrated as closed contours. Each generated ROI was manually reviewed, and any incorrect ROIs were corrected using the built-in graphical user interface of the algorithm. The images with segmented lipid droplets (marked with ROIs) were exported as a ZIP archive and subsequently imported into ImageJ software. After setting the spatial calibration (pixels/µm; based on the microscope’s acquisition parameters), the “Area” and “Feret’s diameter (maximum caliper distance across the droplet at any angle)” parameters (in the “Analyze” tabs of the software) were selected for measurement. Statistical analyses were conducted using GraphPad Prism.

**Flow cytometry**

Effects of LCA on cell death in cultured hepatocytes were determined by a flow cytometry-based method, as described previously^7,26^, with minor modifications. Briefly, cells grown to 70%−80% confluence in a 6-well dish were treated with LCA, followed by washing with 1 mL PBS (pre-heated to 37°C), and then trypsinized with 0.25% trypsin, followed by centrifugation at 1,200 *g* for 5 min at 4°C. A total of 1 × 10^6^ trypsinized cells were washed with 1 mL PBS (pre-cooled to 4°C). Cell suspensions were centrifuged at 1,200 *g* for 5 min at 4°C. The pellets were resuspended with 100 μL Binding Buffer provided in the Annexin V Apoptosis Detection Kit, and then incubated with 5 μL Annexin V-FITC solution and 10 μL propidium iodide (PI) for 10 min at room temperature in the dark. Cell suspensions were then diluted with 400 μL Binding Buffer, and immediately subjected to flow cytometry analysis. Flow cytometry was performed on a BD LSRFortessa X20 cell analyzer, with the 488-nm laser and the 530/30 filter used to excite and detect the fluorescence of Annexin V-FITC, and the 561-nm laser and 610/30 filter PI. Detector voltages were optimized using a modified voltage titration approach.^42^ Gating strategies used during the analysis were: (a) the FSC-H and SSC-H for selecting intact cells, and (b) FSC-H and FSC-A for excluding doublets. Gate boundaries were set either based on control samples, or followed density distributions based on best practices. Data were collected by the FACSDiva software (v8.0.2, BD Biosciences), followed by exporting as the FCS 3.1 format. The numbers of early apoptotic cells (Annexin V-positive and PI-negative populations), and late apoptotic and necroptotic cells (Annexin V-positive and PI-positive populations) were quantified using FlowJo software (v10.4.0, BD Biosciences). For this experiment, a combination of manual gating and computational analysis approaches^43^ was used.

**IB assays**

To analyze the levels of p-AMPKα, p-ACC, p-RPA, and p-H2AX in mouse primary hepatocytes, HepaG2 cells, and HepaRG cells, cells grown to 70%−80% confluence in a well of a 6-well dish were lysed with 250 μL ice-cold Triton lysis buffer. The lysates were then centrifuged at 20,000 *g* for 10 min at 4°C, and an equal volume of 2× SDS sample buffer was added to the supernatant. Samples were then boiled for 10 min and then directly subjected to immunoblotting. To analyze the levels of p-AMPKα and p-ACC in liver tissues, mice were anesthetized after the indicated treatments. Freeze-clamped tissues were immediately lysed with ice-cold Triton lysis buffer (10 μL/mg tissue weight for liver), followed by homogenization and centrifugation as described above. The lysates were then mixed with 2× SDS sample buffer, boiled, and subjected to immunoblotting. All samples were subjected to IB on the same day of preparation, and any freeze–thaw cycles were avoided.

For IB, the SDS-polyacrylamide gels were prepared in house as described previously^34,44,45^. The thickness of the gels used in this study was 1.0 mm. Samples of less than 10 μL were loaded into wells, and the electrophoresis was run at 100 V (by PowerPac HC High-Current Power Supply, Bio-Rad) in a Mini-PROTEAN Tetra Electrophoresis Cell (Bio-Rad). In this study, all samples were resolved on 8% resolving gels, except those for H3, p-H2AX, RPA, and p-RPA proteins, which were on 15% gels. The resolved proteins were then transferred to the PVDF membrane (0.45 μm, IPVH00010; Merck) as described previously. The PVDF membrane was then blocked by 5% (w/v) BSA (for all antibodies against phosphorylated proteins) or 5% (w/v) non-fat milk (for all antibodies against total proteins) dissolved in TBST for 2 h on an orbital shaker at 60 rpm at room temperature, followed by rinsing with TBST twice, 5 min each. The PVDF membrane was then incubated overnight at 4 °C on an orbital shaker at 60 rpm with the desired primary antibody, followed by rinsing with TBST three times, 5 min each at room temperature, and then incubation with the secondary antibodies for 3 h at room temperature with gentle shaking. The secondary antibody was then removed, and the PVDF membrane was further washed three times with TBST, 5 min each at room temperature. The PVDF membrane was incubated in ECL mixture (prepared by mixing equal volumes of ECL solution and peroxide solution) for 5 min, and then exposed to Medical X-Ray Film (FUJIFILM). The films were then developed with X-OMAT MX Developer (Carestream) and X-OMAT MX Fixer and Replenisher solutions (Carestream) on a Medical X-Ray Processor (Carestream) using Developer (Model 002; Carestream). The developed films were scanned with an Epson Perfection V850 Pro scanner (Epson) using Epson Scan software (v.3.9.3.4) and cropped in Photoshop 2023 (Adobe). The levels of total proteins and phosphorylated proteins were analyzed on separate gels, and representative immunoblots are shown.

**Determination of LCA concentrations**

LCA concentrations were determined as described previously^3,30^. In brief, hepatocytes (60%−70% in 10-cm dish, rinsed with PBS and trypsinized), tissues (50 mg liver tissues collected from each mouse after anesthetizing and blood-draining), or serum (50 μL, collected from each mouse or monkey) were vigorously mixed (for serum) or homogenized (for others) with 1 mL of 80% methanol (v/v, in water) containing 100 μg/L CA-d5 and 4 µg/mL [U-^13^C]-glutamine as internal standards. After centrifugation at 15,000 *g* for 15 min at 4°C, 30 µL supernatant was loaded into an injection vial equipped with a snap cap, as described in the “Mouse hyperinsulinemia-euglycemic clamp” section. A total of 2 µL supernatant was injected into an ACQUITY HSS T3 column (Waters). Measurement was performed on a QTRAP 6500 Plus MS (SCIEX) connected to an ACQUITY I-class UPLC system (Waters). The mobile phase consisted of 10 mmol/L ammonium formate containing 0.005% formic acid (v/v) in the LC–MS-grade water (mobile phase A) and LC–MS-grade acetonitrile (mobile phase B) run at a flow rate of 0.4 mL/min. The HPLC gradient was as follows: 30% B for 1 min, then to 100% B at the 10^th^ min, hold for 2 min, and then back to 30% B, hold for another 3 min. MS was performed using a Turbo V ion source in negative mode at a spray voltage of −5,500 V, with source temperature at 550°C, gas NO.1 at 50 psi, gas NO.2 at 60 psi, curtain gas at 35 psi, and collision gas at “medium”. Compounds were measured using the MRM mode, and declustering potentials and collision energies were optimized using analytical standards. The following transitions (Q1/Q3) were used for monitoring each compound: 375.2/375.2 for LCA; 149.9/114 for [U-^13^C]-glutamine; and 412.3/348.3 for CA-d5. For quantification of LCA, the LCA-d4 dissolved in individual lysates were used to generate corresponding standard curves by plotting the area ratios of labelled LCA (areas of LCA divided by the two ISs) against the added/actual concentrations of labeled LCA. To calculate the concentrations of LCA (in µmol/L) in the liver, the average density of mouse liver, 1.065 g/mL (ref. ^46^), was used.

**Measurement of NAD^+^**

The levels of NAD^+^ were determined as described previously^16,17^, with minor modifications. In brief, 50 mg mouse liver tissues were homogenized in 1 mL of 80% methanol (v/v in water, containing 4 µg/mL [U-^13^C]-glutamine as an internal standard). After centrifugation at 15,000 *g* for another 15 min at 4°C, 800 μL aqueous phase was collected and lyophilized as described in the “Mouse hyperinsulinemia-euglycemic clamp” section, and then dissolved in 50 μL of 50% (v/v, in water) acetonitrile, followed by centrifugation at 15,000 *g* for another 30 min at 4 °C. A total of 20 µL supernatant was loaded into an injection vial equipped with a snap cap, followed analyses of NAD^+^ levels by the QTRAP 5500 MS as described in the “Mouse hyperinsulinemia-euglycemic clamp” section. Data were collected using Analyst software (v.1.7.1, SCIEX), and the relative amounts of metabolites were analyzed using MultiQuant software (v.3.0.3, SCIEX). Levels of NAD^+^ were measured using full scan mode with m/z values 662.0/540.1, and 149.9/114 for [U-^13^C]-glutamine. Data were collected using Analyst software (v.1.7.1, SCIEX), and the relative amounts of metabolites were analyzed using MultiQuant software (v.3.0.3, SCIEX).

**Statistical** **analysis**

Statistical analyses were performed using Prism 10 (GraphPad Software). Each group of data was subjected to Kolmogorov-Smirnov test, Anderson-Darling test, D’Agostino-Pearson omnibus test, or Shapiro-Wilk test for normal distribution when applicable. An unpaired two-sided Student’s *t*-test was used to determine the significance between two groups of normally distributed data. Welch’s correction was used for groups with unequal variances. For comparison between multiple groups with two fixed factors, an ordinary two-way ANOVA or two-way repeated-measures (RM) ANOVA (for blood glucose measured during oGTT, ipGTT, ITT, and clamping) was used, followed by Tukey’s or Sidak’s multiple comparisons test as specified in the legends. The assumptions of homogeneity of error variances were tested using F-test (*P* > 0.05). Geisser-Greenhouse’s correction was used where applicable. The adjusted means and SEM, or s.d., were recorded when the analysis met the above standards. Differences were considered significant when *P* < 0.05, or *P* > 0.05 with large differences of observed effects (as suggested in refs. ^47,48^).

**Supplementary references**

1	Nguyen, L. N. *et al.* Mfsd2a is a transporter for the essential omega-3 fatty acid docosahexaenoic acid. *Nature* **509**, 503-506 (2014). <https://doi.org:10.1038/nature13241>

2	Zhang, C. S. *et al.* Identification of serum metabolites enhancing inflammatory responses in COVID-19. *Sci China Life Sci* **65**, 1971-1984 (2022). <https://doi.org:10.1007/s11427-021-2099-7>

3	Qu, Q. *et al.* Lithocholic acid phenocopies anti-ageing effects of calorie restriction. *Nature* (2024). <https://doi.org:10.1038/s41586-024-08329-5>

4	Zhang, C.-S. *et al.* Fructose-1,6-bisphosphate and aldolase mediate glucose sensing by AMPK. *Nature* **548**, 112-116 (2017). <https://doi.org:10.1038/nature23275>

5	Zong, Y. *et al.* Hierarchical activation of compartmentalized pools of AMPK depends on severity of nutrient or energy stress. *Cell Res* **29**, 460-473 (2019). <https://doi.org:10.1038/s41422-019-0163-6>

6	Zhang, C. S. *et al.* The aldolase inhibitor aldometanib mimics glucose starvation to activate lysosomal AMPK. *Nat Metab* (2022). <https://doi.org:10.1038/s42255-022-00640-7>

7	Hu, H.-H. *et al.* Glucose starvation mimetic aldometanib removes immune barriers permitting mice with hepatocellular carcinoma to live to normal ages. *Cell research* (2025). <https://doi.org:10.1038/s41422-025-01195-4>

8	Brunetti-Pierri, N. *et al.* Balloon Catheter Delivery of Helper-dependent Adenoviral Vector Results in Sustained, Therapeutic hFIX Expression in Rhesus Macaques. *Molecular Therapy* **20**, 1863-1870 (2012). <https://doi.org:https://doi.org/10.1038/mt.2012.143>

9	Powell, C. J., Kapeghian, J. C., Bernal, J. C. & Foster, J. R. Hepatitis A Virus Infection in Cynomolgus Monkeys Confounds the Safety Evaluation of a Drug Candidate. *Int J Toxicol* **43**, 368-376 (2024). <https://doi.org:10.1177/10915818241237992>

10	Qu, P. *et al.* DT-109 ameliorates nonalcoholic steatohepatitis in nonhuman primates. *Cell metabolism* **35**, 742-757.e710 (2023). <https://doi.org:https://doi.org/10.1016/j.cmet.2023.03.013>

11	Sun, S. *et al.* CHIT1-positive microglia drive motor neuron ageing in the primate spinal cord. *Nature* **624**, 611-620 (2023). <https://doi.org:10.1038/s41586-023-06783-1>

12	Esquejo, R. M. *et al.* Activation of Liver AMPK with PF-06409577 Corrects NAFLD and Lowers Cholesterol in Rodent and Primate Preclinical Models. *EBioMedicine* **31**, 122-132 (2018). <https://doi.org:10.1016/j.ebiom.2018.04.009>

13	Zhao, P. *et al.* An AMPK–caspase-6 axis controls liver damage in nonalcoholic steatohepatitis. *Science* **367**, 652-660 (2020). <https://doi.org:doi:10.1126/science.aay0542>

14	Morrow, M. R. *et al.* Inhibition of ATP-citrate lyase improves NASH, liver fibrosis, and dyslipidemia. *Cell metabolism* **34**, 919-936 e918 (2022). <https://doi.org:10.1016/j.cmet.2022.05.004>

15	Gautam, J. *et al.* ACLY inhibition promotes tumour immunity and suppresses liver cancer. *Nature* **645**, 507-517 (2025). <https://doi.org:10.1038/s41586-025-09297-0>

16	Li, M. *et al.* Transient Receptor Potential V Channels Are Essential for Glucose Sensing by Aldolase and AMPK. *Cell metabolism* **30**, 508-524 e512 (2019). <https://doi.org:10.1016/j.cmet.2019.05.018>

17	Li, M. *et al.* AMPK-PDZD8-GLS1 axis mediates calorie restriction-induced lifespan extension. *Cell Res* (2024). <https://doi.org:10.1038/s41422-024-01021-3>

18	Woods, A. *et al.* Liver-Specific Activation of AMPK Prevents Steatosis on a High-Fructose Diet. *Cell reports* **18**, 3043-3051 (2017). <https://doi.org:10.1016/j.celrep.2017.03.011>

19	Garcia, D. *et al.* Genetic Liver-Specific AMPK Activation Protects against Diet-Induced Obesity and NAFLD. *Cell reports* **26**, 192-208 e196 (2019). <https://doi.org:10.1016/j.celrep.2018.12.036>

20	Parks, J. S., Johnson, F. L., Wilson, M. D. & Rudel, L. L. Effect of fish oil diet on hepatic lipid metabolism in nonhuman primates: lowering of secretion of hepatic triglyceride but not apoB. *Journal of lipid research* **31**, 455-466 (1990).

21	Wang, J. *et al.* Targeting ANGPTL3 by GalNAc-conjugated siRNA ANGsiR10 lowers blood lipids with long-lasting and potent efficacy in mice and monkeys. *Mol Ther Nucleic Acids* **31**, 68-77 (2023). <https://doi.org:10.1016/j.omtn.2022.11.023>

22	Zhang, C. S. *et al.* Metformin Activates AMPK through the Lysosomal Pathway. *Cell metabolism* **24**, 521-522 (2016). <https://doi.org:10.1016/j.cmet.2016.09.003>

23	Li, M. *et al.* AMPK targets PDZD8 to trigger carbon source shift from glucose to glutamine. *Cell Res* (2024). <https://doi.org:10.1038/s41422-024-00985-6>

24	Zhang, C.-J. *et al.* TRPV4 acetylation in prenatal liver prevents low glucose-induced inhibition of mTORC1 and safeguards fetal development. *Vita*, null-null

25	Cheratta, A. R. *et al.* Caspase cleavage and nuclear retention of the energy sensor AMPK-α1 during apoptosis. *Cell reports* **39**, 110761 (2022). <https://doi.org:https://doi.org/10.1016/j.celrep.2022.110761>

26	Wu, Y. Q. *et al.* Low glucose metabolite 3-phosphoglycerate switches PHGDH from serine synthesis to p53 activation to control cell fate. *Cell Res* (2023). <https://doi.org:10.1038/s41422-023-00874-4>

27	Zhang, T. *et al.* Metabolic orchestration of cell death by AMPK-mediated phosphorylation of RIPK1. *Science* **380**, 1372-1380 (2023). <https://doi.org:doi:10.1126/science.abn1725>

28	McCurdy, C. E. *et al.* Maternal high-fat diet triggers lipotoxicity in the fetal livers of nonhuman primates. *J Clin Invest* **119**, 323-335 (2009). <https://doi.org:10.1172/jci32661>

29	Chu, M. P. *et al.* Intrahepatic lipid, not visceral or muscle fat, is correlated with insulin resistance in older, female rhesus macaques. *Obesity* **21**, 2021-2028 (2013). <https://doi.org:10.1002/oby.20339>

30	Qu, Q. *et al.* Lithocholic acid binds TULP3 to activate sirtuins and AMPK to slow down ageing. *Nature* (2024). <https://doi.org:10.1038/s41586-024-08348-2>

31	Shiota, M. Measurement of glucose homeostasis in vivo: combination of tracers and clamp techniques. *Methods Mol Biol* **933**, 229-253 (2012). <https://doi.org:10.1007/978-1-62703-068-7_15>

32	Hui, X. *et al.* Adipocyte SIRT1 controls systemic insulin sensitivity by modulating macrophages in adipose tissue. *EMBO reports* **18**, 645-657 (2017). <https://doi.org:10.15252/embr.201643184>

33	Zhang, Y., Xu, L., Liu, X. & Wang, Y. Evaluation of insulin sensitivity by hyperinsulinemic-euglycemic clamps using stable isotope-labeled glucose. *Cell Discov* **4**, 17 (2018). <https://doi.org:10.1038/s41421-018-0016-3>

34	Ma, T. *et al.* Low-dose metformin targets the lysosomal AMPK pathway through PEN2. *Nature* (2022). <https://doi.org:10.1038/s41586-022-04431-8>

35	Kleiner, D. E. *et al.* Design and validation of a histological scoring system for nonalcoholic fatty liver disease. *Hepatology* **41**, 1313-1321 (2005). <https://doi.org:10.1002/hep.20701>

36	Zhang, Y. L. *et al.* AMP as a low-energy charge signal autonomously initiates assembly of AXIN-AMPK-LKB1 complex for AMPK activation. *Cell Metab.* **18**, 546-555 (2013). <https://doi.org:10.1016/j.cmet.2013.09.005>

37	He, B. *et al.* Identification of cytochrome b5 CYTB-5.1 and CYTB-5.2 in C. elegans; evidence for differential regulation of SCD. *Biochim Biophys Acta Mol Cell Biol Lipids* **1863**, 235-246 (2018). <https://doi.org:10.1016/j.bbalip.2017.12.007>

38	Li, M. *et al.* Aldolase is a sensor for both low and high glucose, linking to AMPK and mTORC1. *Cell Res* (2020). <https://doi.org:10.1038/s41422-020-00456-8>

39	Li, M. *et al.* Hierarchical inhibition of mTORC1 by glucose starvation-triggered AXIN lysosomal translocation and by AMPK. *Life Metabolism* (2023). <https://doi.org:10.1093/lifemeta/load005>

40	Zhang, C.-S. & Lin, S.-C. in *Nutrient Sensing in Eukaryotes*	(eds Fiona M. Russell & D. Grahame Hardie) 105-119 (Springer US, 2025).

41	Stringer, C., Wang, T., Michaelos, M. & Pachitariu, M. Cellpose: a generalist algorithm for cellular segmentation. *Nature methods* **18**, 100-106 (2021). <https://doi.org:10.1038/s41592-020-01018-x>

42	Perfetto, S. P., Ambrozak, D., Nguyen, R., Chattopadhyay, P. K. & Roederer, M. Quality assurance for polychromatic flow cytometry using a suite of calibration beads. *Nature protocols* **7**, 2067-2079 (2012). <https://doi.org:10.1038/nprot.2012.126>

43	Liechti, T. *et al.* An updated guide for the perplexed: cytometry in the high-dimensional era. *Nat Immunol* **22**, 1190-1197 (2021). <https://doi.org:10.1038/s41590-021-01006-z>

44	Zhang, C. S., Li, M. & Lin, S. C. Methods to Study Lysosomal AMPK Activation. *Methods in enzymology* **587**, 465-480 (2017). <https://doi.org:10.1016/bs.mie.2016.09.071>

45	Zhang, C. S., Li, M., Zong, Y. & Lin, S. C. Determining AMPK Activation via the Lysosomal v-ATPase-Ragulator-AXIN/LKB1 Axis. *Methods Mol Biol* **1732**, 393-411 (2018). <https://doi.org:10.1007/978-1-4939-7598-3_25>

46	Proto, A. V. & Lane, E. J. Visualization of differences in soft-tissue densities. The liver in ascites. *Radiology* **121**, 19-23 (1976). <https://doi.org:10.1148/121.1.19>

47	Amrhein, V., Greenland, S. & McShane, B. Scientists rise up against statistical significance. *Nature* **567**, 305-307 (2019). <https://doi.org:10.1038/d41586-019-00857-9>

48	Wasserstein, R. L., Schirm, A. L. & Lazar, N. A. Moving to a World Beyond “p < 0.05”. *The American Statistician* **73**, 1-19 (2019). <https://doi.org:10.1080/00031305.2019.1583913>
