# Supplementary material for: Lithocholic acid alleviates fatty liver in mice and non-human primate macaques
Source: Life Metab. 2026 Jul 23;5(6):loag023. doi: 10.1093/lifemeta/loag023 (PMC13591641; doi:10.1093/lifemeta/loag023)
Supplement: Supplementary file 1 [file loag023_supplementary_data.zip › Supplementary_Fig1-6.pdf]

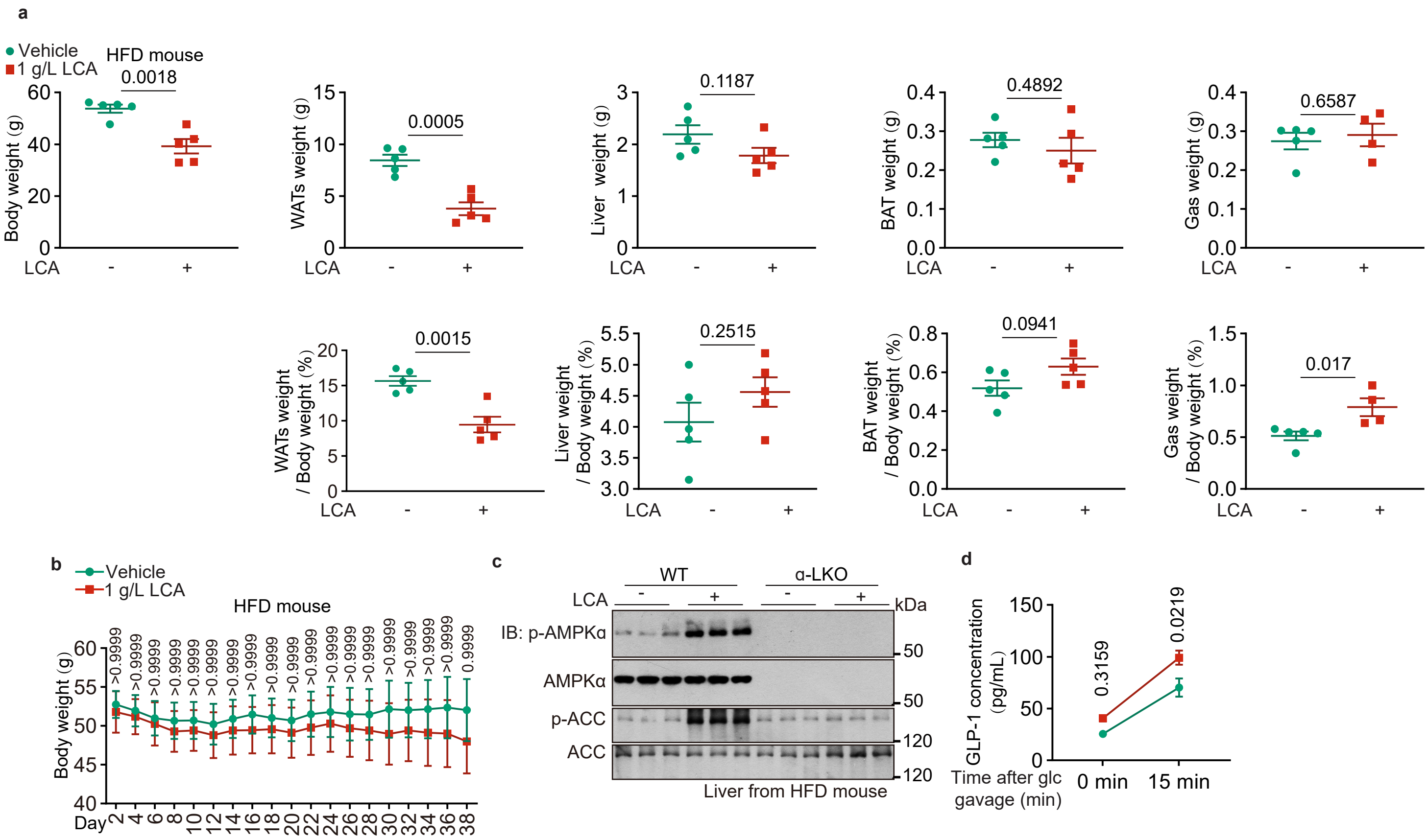

Supplementary Fig 1

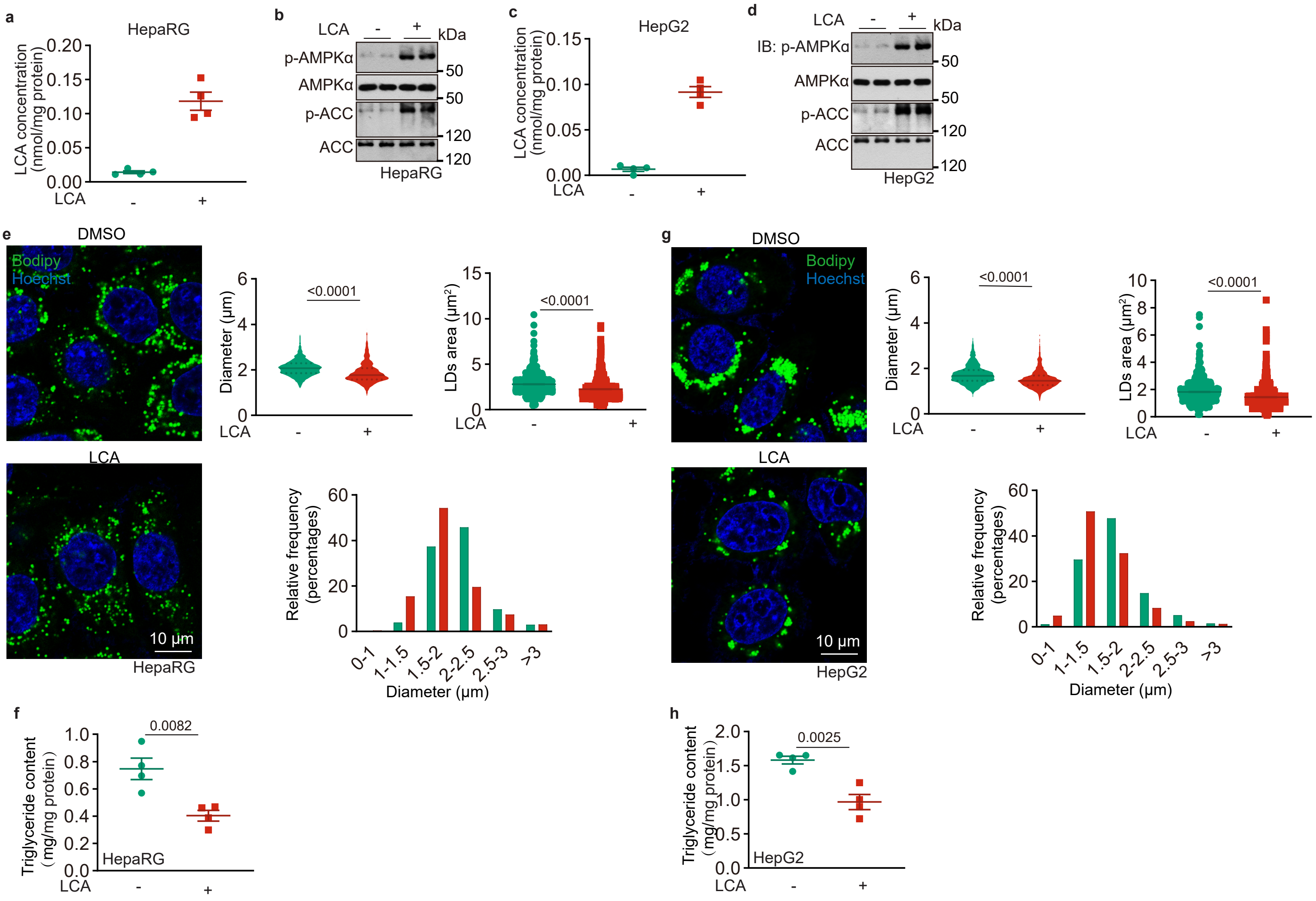

Supplementary Fig 2

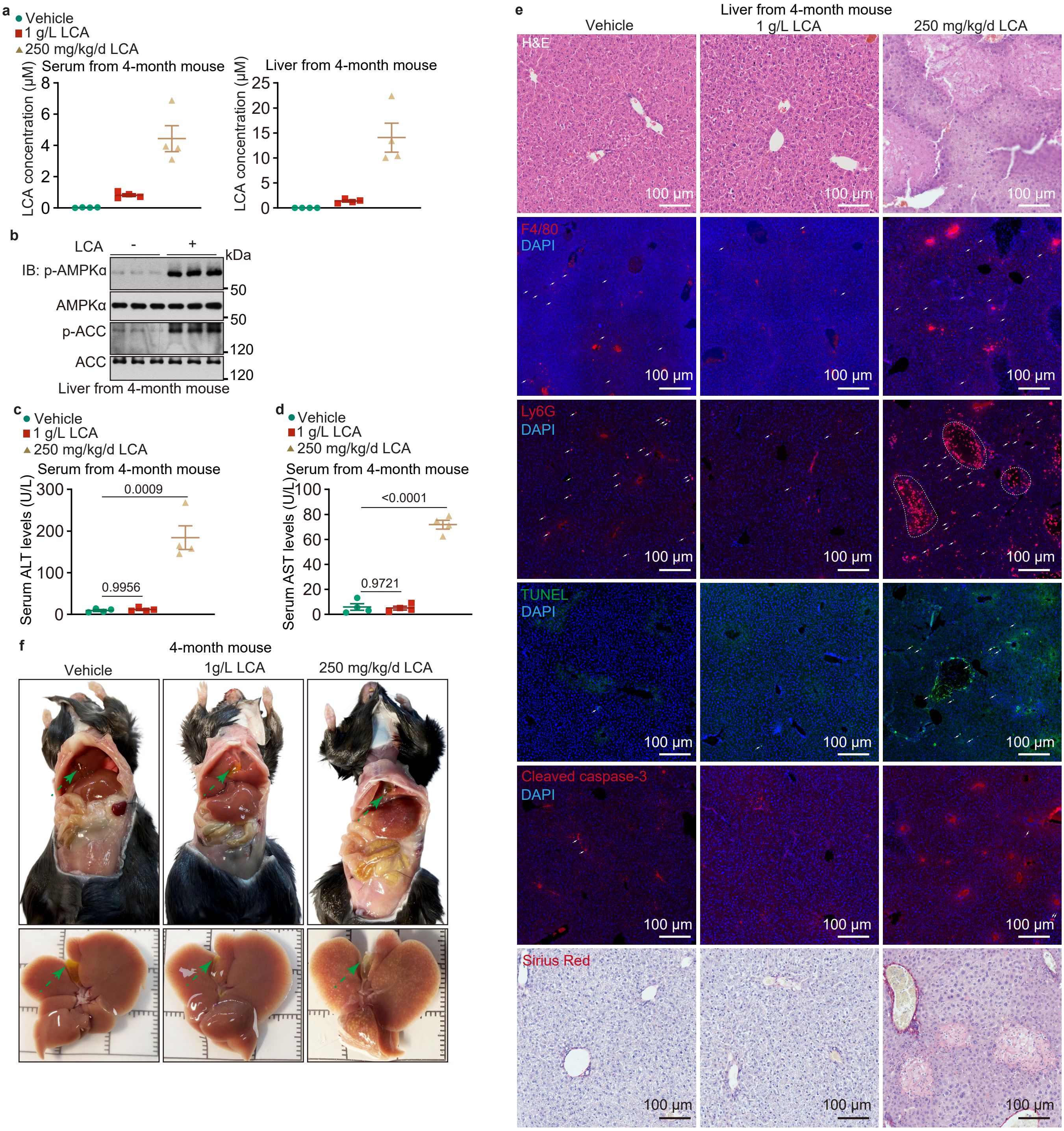

Supplementary Fig 3

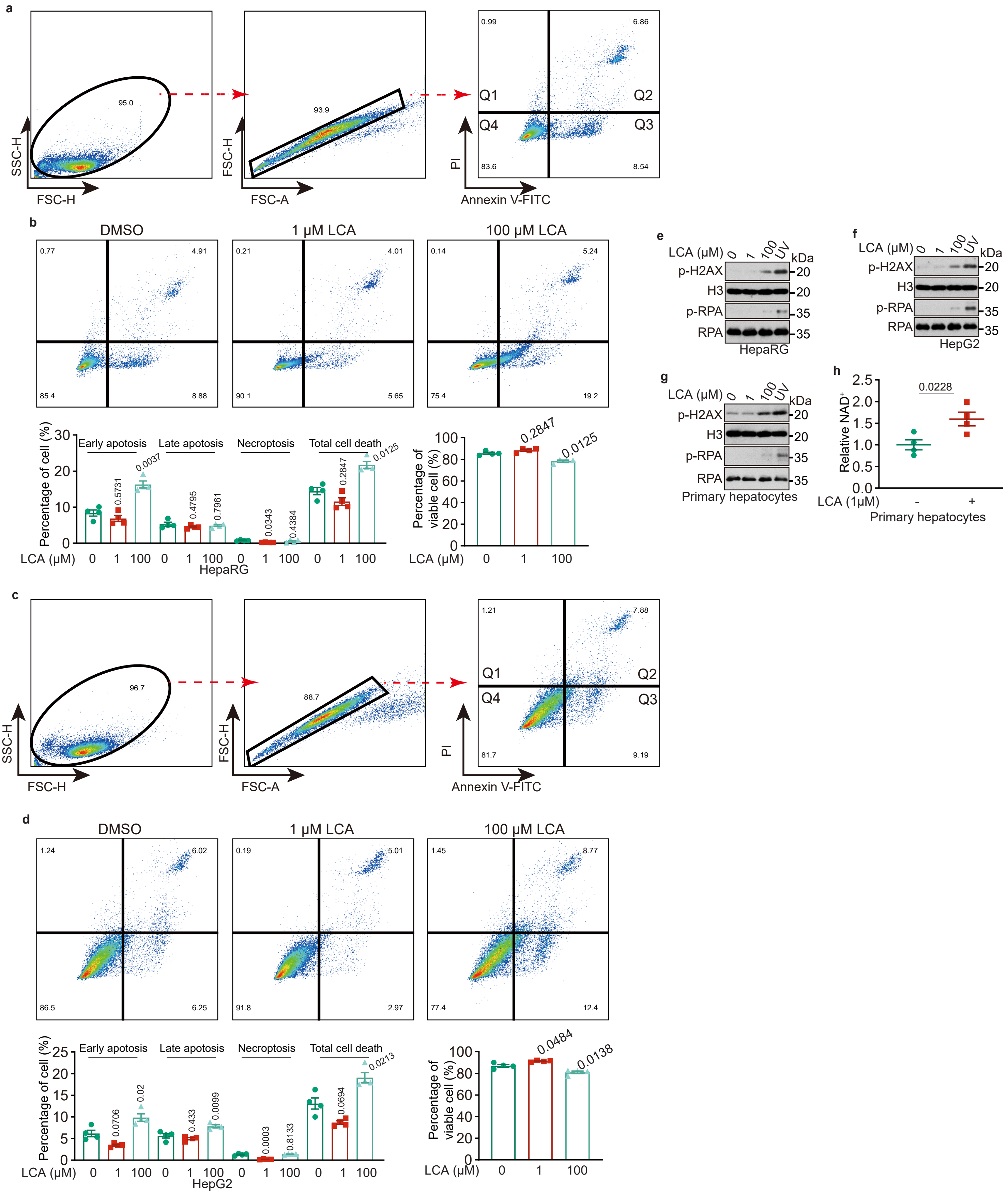

Supplementary Fig 4

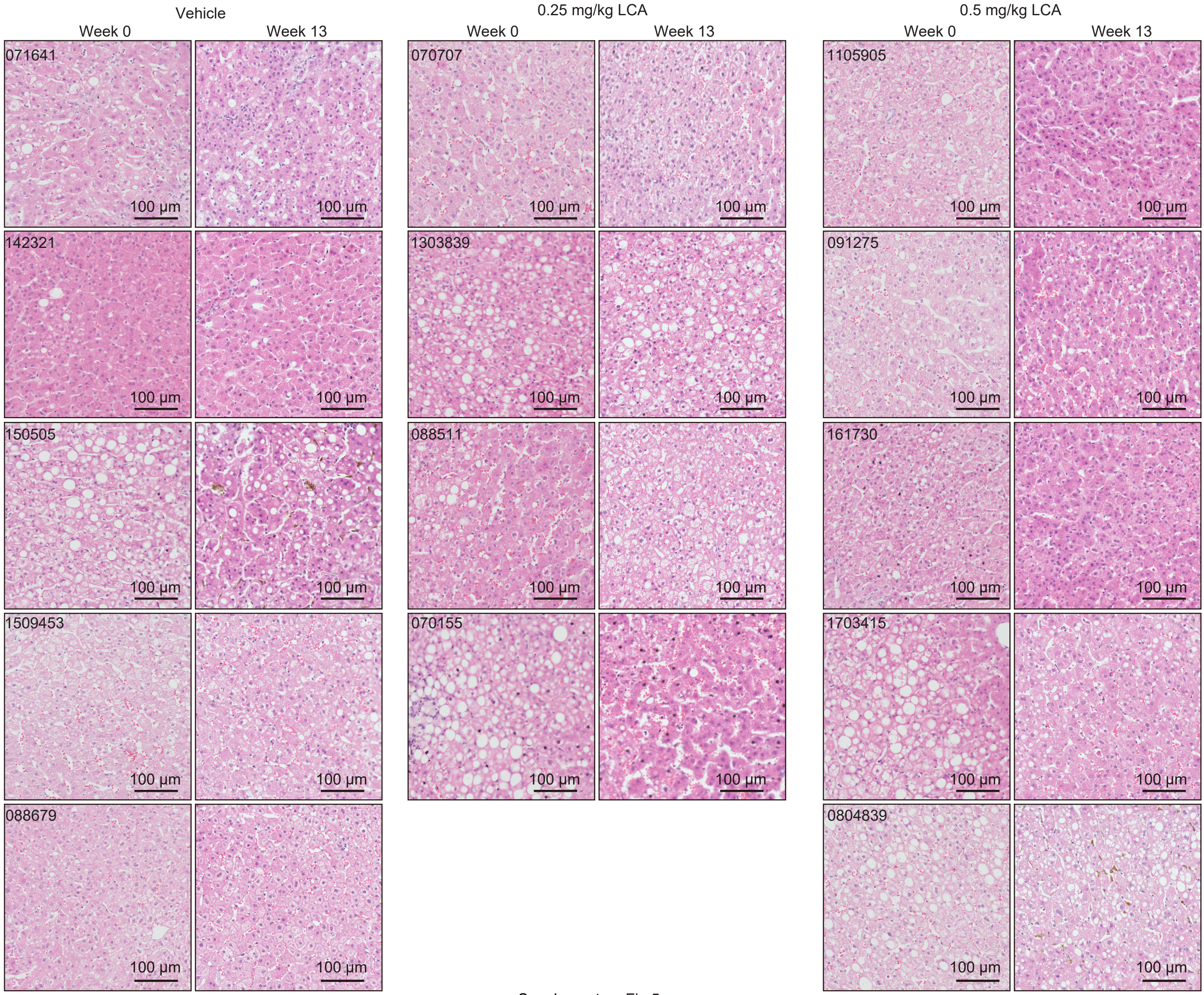

Supplementary Fig 5

Vehicle

Week 0 Week 13

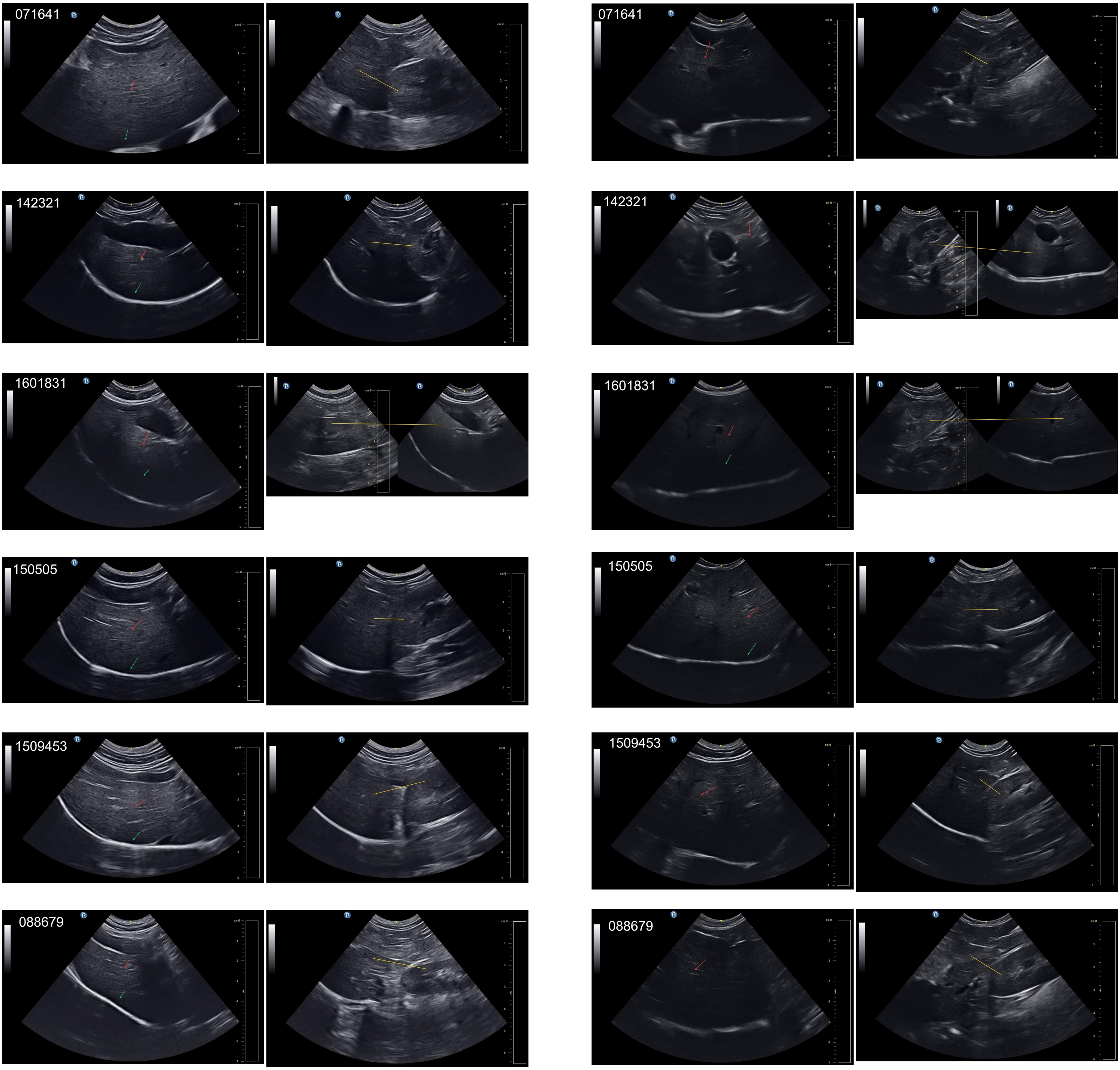

Supplementary Fig 6 Cont.

0.25 mg/kg LCA

Week 0 | Week 13

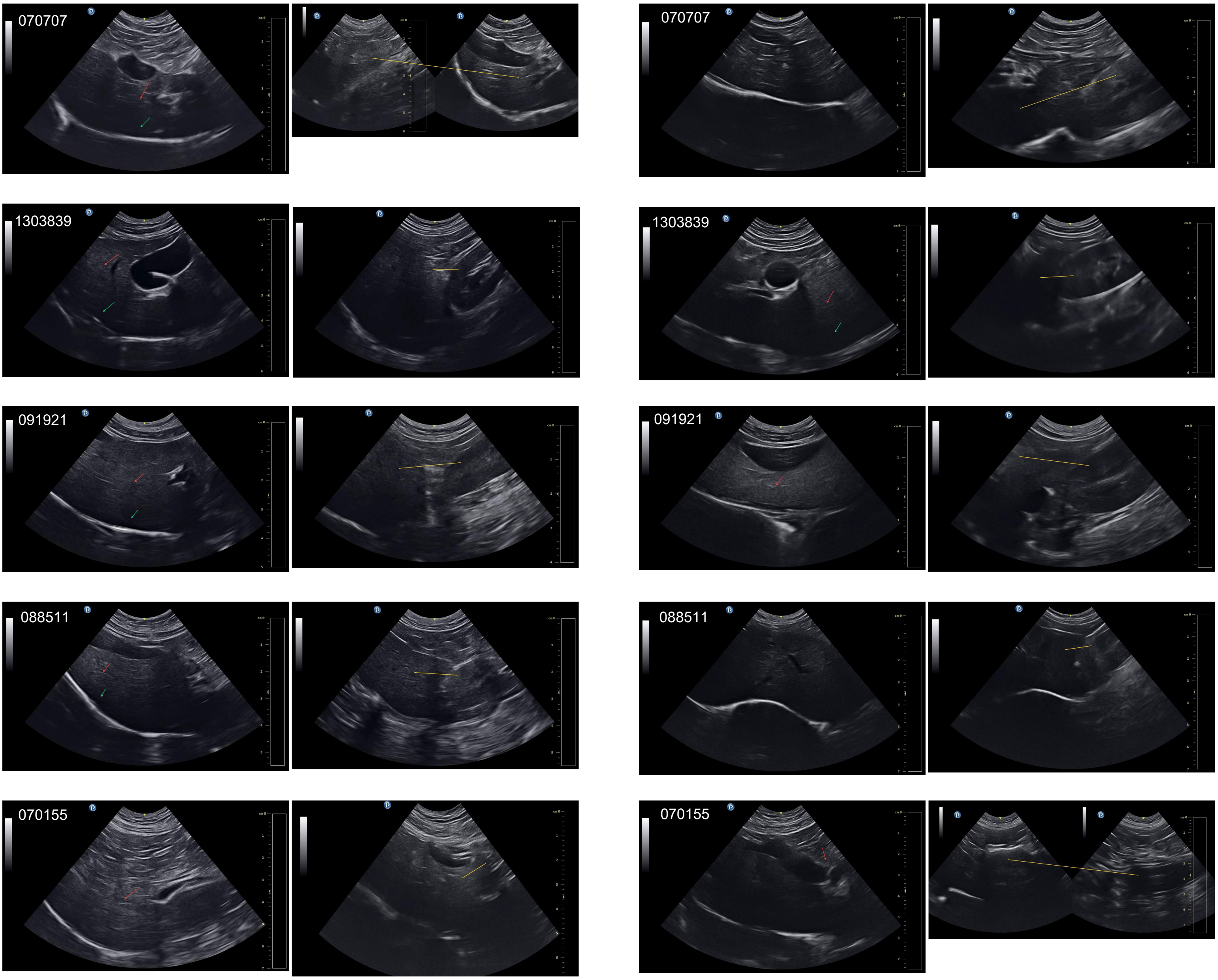

Supplementary Fig 6 Cont.

0.5 mg/kg LCA

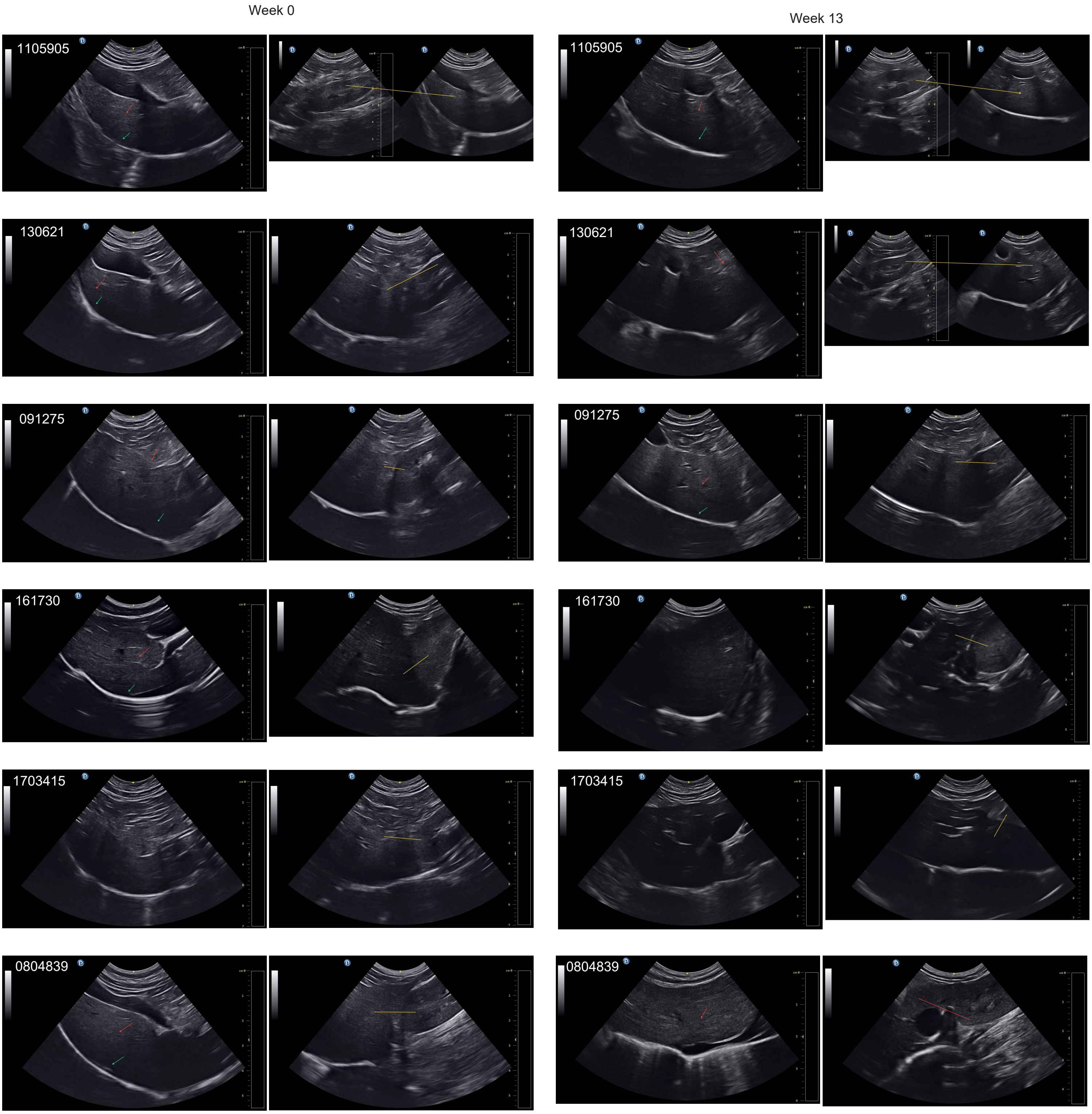

Supplementary Fig 6
