# Supplementary material for: Lithocholic acid alleviates fatty liver in mice and non-human primate macaques
Source: Life Metab. 2026 Jul 23;5(6):loag023. doi: 10.1093/lifemeta/loag023 (PMC13591641; doi:10.1093/lifemeta/loag023)

Fig 1d

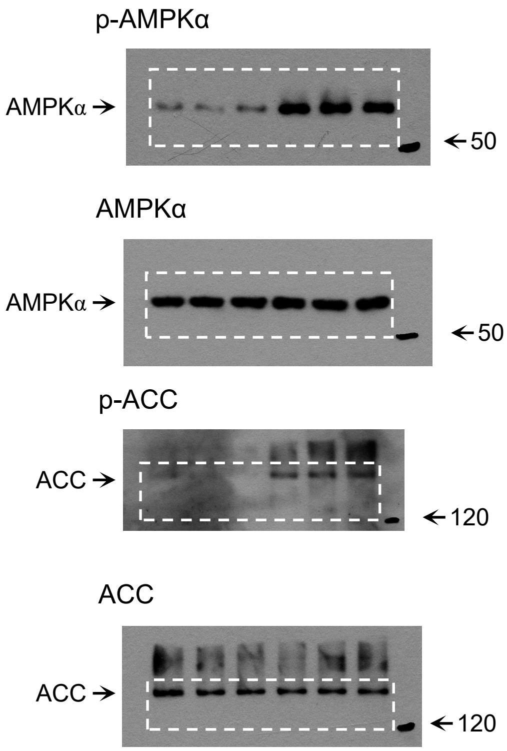

Fig 2e

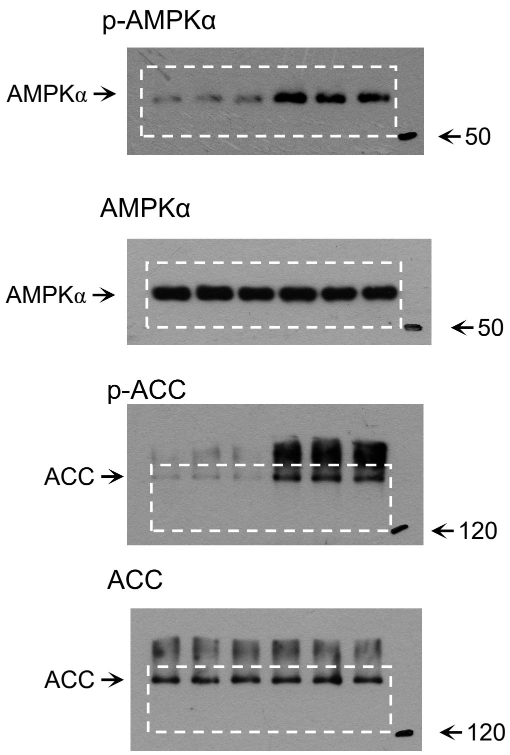

Extended data , Fig. 1c

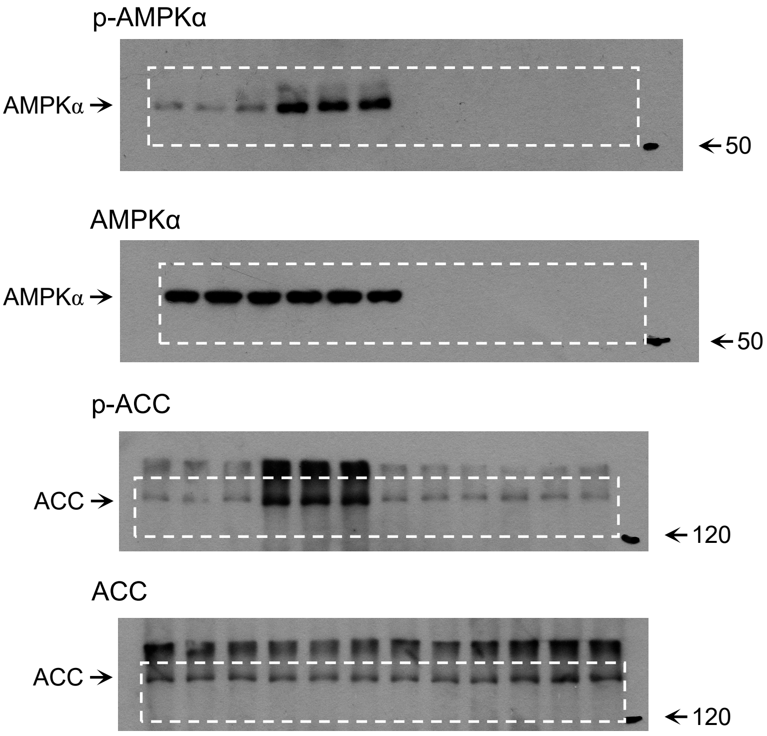

Extended data , Fig. 2b

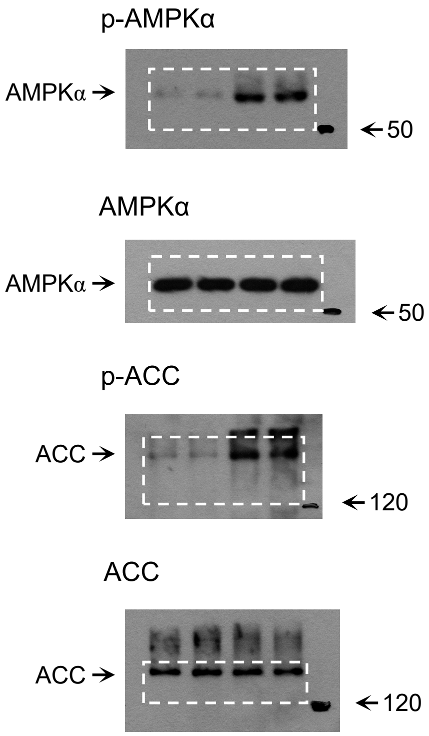

Extended data , Fig. 2d

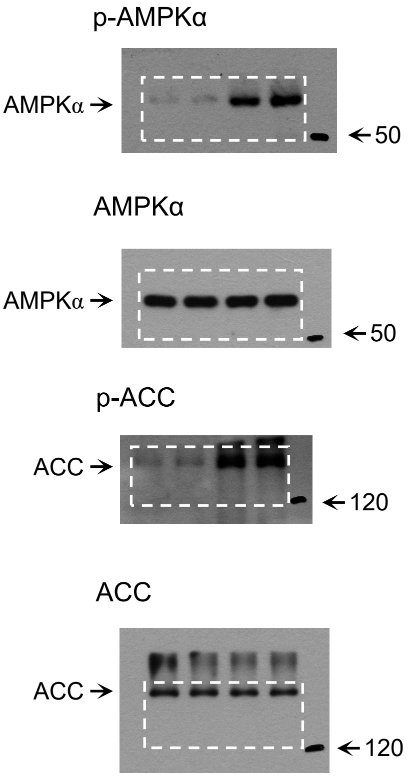

Extended data , Fig. 3b

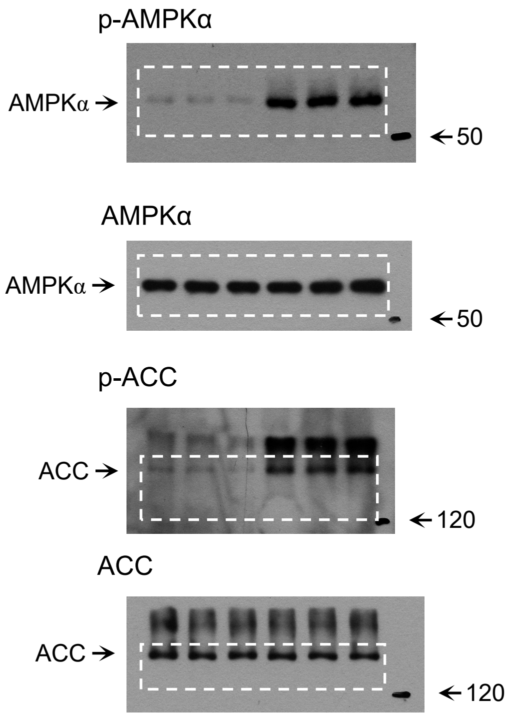

Extended data , Fig. 4e

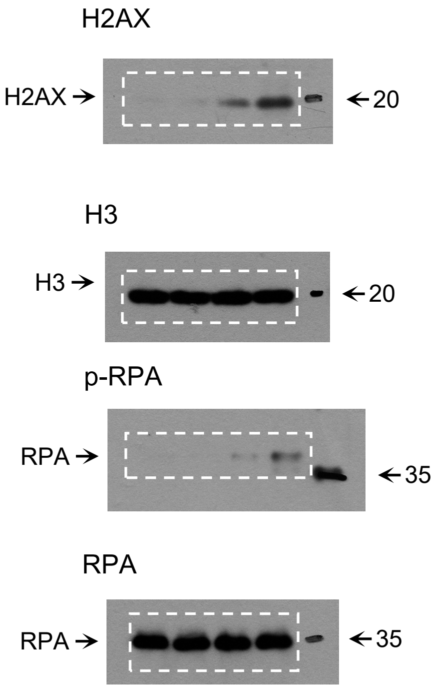

Extended data , Fig. 4f

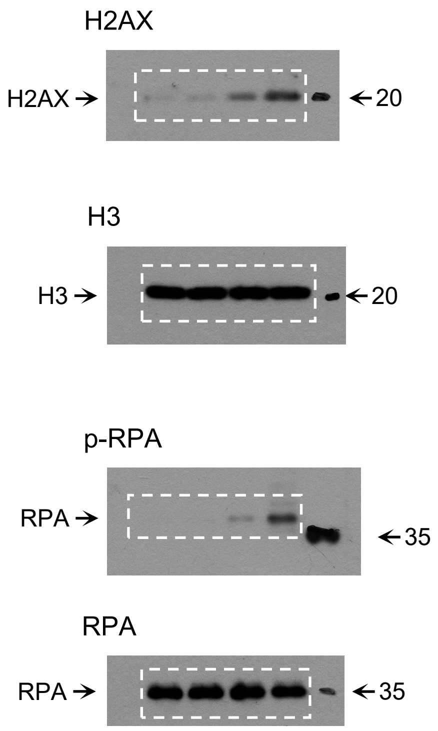

Extended data , Fig. 4g

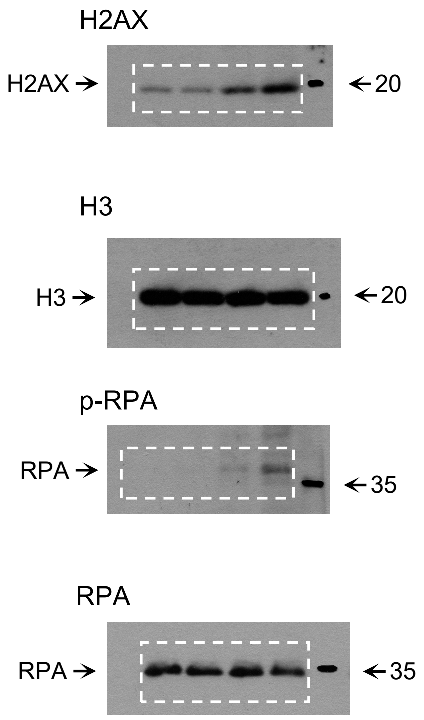

Supplement: Supplementary file 1 [file loag023_supplementary_data.zip › Fig_S7.pdf]
