# Supplementary material for: Lithocholic acid alleviates fatty liver in mice and non-human primate macaques
Source: Life Metab. 2026 Jul 23;5(6):loag023. doi: 10.1093/lifemeta/loag023 (PMC13591641; doi:10.1093/lifemeta/loag023)
Supplement: Supplementary file 1 [file loag023_supplementary_data.zip › Figure_legends_of_Supplementary_Fig1-6.docx]

**Figure** **S1** LCA alleviates high-fat diet-induced fatty liver in mice

(a, b) LCA reduces fat content in HFD-fed mice. Mice were fed with HFD to induce fatty liver, and then treated with (2-hydroxypropyl)-β-cyclodextrin-coated LCA at 1 g/L in drinking water, which results in exposure levels similar to those found in the serum and liver of calorie-restricted mice (as shown in Fig. 1a-c), over a period of 1 month. During the treatment, the body weights of the mice were measured every other day (b). At the end of the treatment, body weights, as well as the weights of white adipose tissues (WATs; the sum of inguinal, perirenal, gonadal, and mesenteric white adipose tissue weights), liver, brown adipose tissue (BAT), and gastrocnemius (Gas) were determined (a). Additionally, tissue/organ-to-body weight ratios were calculated (a). Data are mean ± SEM; n = 4 (b, and the weights of gastrocnemius in the LCA-treated group in a) or 5 (a) mice for each condition, with P values calculated by two-sided Student’s *t*-test (a), or by two-way ANOVA, followed by Sidak (b).

(c) Validation of AMPKα knockout efficiency in the livers of *AMPKα*-LKO mice. The *AMPKα*-LKO (α-LKO) mice and wildtype (WT) littermates, both fed with HFD for 5 months, were treated with LCA as in Fig. 1k. The expression of AMPKα in liver tissues, along with the activation of AMPK, was determined by immunoblotting.

(d) LCA promotes postprandial GLP-1 secretion. Wildtype mice were fed with HFD for 5 months to induce fatty liver, and were treated with LCA as in Fig. 1b for 2 week. Mice were then fasted for 16 h, followed by gavaging with 2 g/L glucose, and then sacrificed at the indicated time intervals following the glucose gavage. Levels of GLP-1 (total levels, including full-length and two truncated forms) in the plasma were measured. Results are shown as mean ± SEM; n = 4 mice for each time point, and P values by two-way ANOVA, followed by Tukey, all compared to the fasting group.

**Figure** **S2** LCA decreases lipid accumulation in hepatocytes

(a-d) LCA activates AMPK in cultured hepatocytes. The primary-like human HepaRG hepatocytes (a, b) and human hepatoma HepG2 cells (c, d) were treated with 1 µM LCA in the culture media for 4 h, followed by determination of intracellular concentrations of LCA (a, c; data are mean ± SEM; n = 4 dishes of cells for each treatment) and AMPK activity (b, d).

(e-h) LCA decreases lipid accumulation in hepatocytes. HepaRG cells (e, f) and HepG2 cells (g, h) were treated with 250 μM oleic acid (OA) for 12 h, followed by incubation with 1 μM LCA for an additional 4 h. The triglyceride contents (f, h; results are mean ± SEM; n = 4 dishes of cells for each condition, with P value calculated by two-sided Student’s *t*-test) were determined, and the morphology of the lipid droplets, after staining with BODIPY dye (e, g), was examined. In panels e and g, representative images (left panels) and quantification of the total area and size (measured as average diameters and the percentages/distributions of each diameter range) of lipid droplets (right panels, results are mean ± SEM; n = 1128-1306 lipid droplets from 18-25 cells for each condition, with P value analyzed using two-sided Student’s *t*-test with Welch’s correction (average diameters and area in e) or two-sided Student’s *t*-test (others)) are shown.

**Figure** **S3** LCA at CR-mimetic doses does not cause liver damage. Lean mice of 4 months old, were administered the CR-mimetic, 1 g/L LCA in drinking water for 1 month, or received intraperitoneal injections of 250 mpk LCA for 4 consecutive days as a positive control to induce liver damage. Following these treatments, serum and liver samples were collected for analysis. The concentrations of LCA in the serum and livers (a), and the activation of AMPK (b) in the livers were determined. Liver damage indices, including serum ALT and AST levels (c, d), were determined. In addition, liver sections were analyzed for morphology (e, “H&E”), fibrosis (e, “Sirius Red”), macrophage (e, “F4/80”) and neutrophil (e, “Ly6G”) infiltration, and the presence of apoptotic hepatocytes (e, “TUNEL” and “cleaved caspase-3”). Cells with positive staining for each marker are indicated by white arrows. Representative images of the liver are shown in panel (f), with the gallbladder indicated by green arrows. Results are mean ± SEM; n = 4 mice for each condition, with P value calculated by two-way ANOVA, followed by Tukey.

**Figure** **S4** LCA at CR-mimetic dose does not cause cell death or DNA damage in hepatocytes

(a-d) LCA does not cause cell death in cultured hepatocytes. HepaRG (a, b) and HepG2 (c, d) cells were treated with the CR-mimetic, 1 μM LCA, or 100 μM LCA as a positive control to induce cell death, for 12 h. Cell death was assessed using Annexin V-PI staining followed by flow cytometry. During the analysis, intact cells from each sample were selected by FSC-H and SSC-H (left panels of a and c, using a linear scale), followed by FSC-H and FSC-A to exclude doublets (middle panels of a and c, using a linear scale). The fluorescence intensities of propidium iodide (PI) and Annexin V-FITC were then determined and presented as density plots (right panels of a and c, using a logarithmic scale). The plot is divided into four quadrants (Q1, Q2, Q3, and Q4), in which Q1 (Annexin V negative and PI positive populations) represents necroptotic cells, Q2 (Annexin V positive and PI positive populations) represents late apoptotic cells, and Q3 (Annexin V positive and PI negative populations) represents early apoptotic cells. The percentages of early apoptotic, late apoptotic, and necroptotic cells are shown at the bottom of panels of b and d as means ± SEM; n = 4 dishes of cells, with *P* values calculated by two-way ANOVA, followed by Tukey. See also representative density plots on the top of panels of b and d.

(e-g) CR-mimetic LCA does not cause DNA damage in hepatocytes. HepaRG cells (e), HepG2 cells (f), and primary hepatocytes (g) were treated with 1 μM or 100 μM LCA for 12 h, or exposed to 75 J/m^2^ UV followed with incubation in fresh DMEM for another 2 h as a control for DNA damage. Cells were then lysed, followed by determination of the levels of H2AX and RPA32 phosphorylation by immunoblotting.

(h) LCA elevates NAD^+^ levels in hepatocytes. Primary hepatocytes were treated with 1 μM LCA for 24 h, followed by determination of NAD^+^ levels. Results are mean ± SEM; n = 4 dishes of cells for each condition, with P value calculated by two-sided Student’s *t*-test.

**Figure S5** LCA reduces fatty liver in macaques. Monkeys were fed HFD to induce fatty liver, followed by LCA (coated with phospholipids and dissolved in fish oil) gavage at doses of 0.25 mpk (middle panels) and 0.5 mpk (right panels), or vehicle (left panels), twice a day for 13 weeks. Representative images of liver sections for each individual monkey (with the identification numbers of the monkeys labeled) before and after the 13-week LCA administration are shown.

**Figure** **S6** LCA decreases liver echogenicity in macaques. Monkeys were fed HFD to induce fatty liver, followed by LCA gavaging for 13 weeks (as in Fig. 4a). Liver ultrasound images were taken for assessing the severity of fatty liver. Representative images for each individual monkey before and after the 13-week LCA administration are shown. In each image, red arrows point to the near field (the region relatively close to the probe marker) and the green arrow indicates the far field (the region relatively distant from the probe marker). The degree of posterior acoustic attenuation was then evaluated by comparing the brightness between these two fields. If the far field appears darker than the near field and a larger darkened area is observed, the hepatic steatosis is more severe. Additionally, the yellow line segment connects the liver (at one end) to the renal cortex (at the other end; provides an internal reference for echogenicity). A liver echogenicity lower than that of the renal cortex suggests a healthy liver, whereas an echogenicity that is equal to or greater than that of the renal cortex indicates fatty liver. See also Supplementary Table 4 for the severity grades evaluated based on the ultrasound images of each monkey.
